# Three Dimensional Printing and Its Applications Focusing on Microneedles for Drug Delivery

**DOI:** 10.3390/pharmaceutics15061597

**Published:** 2023-05-25

**Authors:** Suhair S. Al-Nimry, Rawand M. Daghmash

**Affiliations:** Department of Pharmaceutical Technology, Faculty of Pharmacy, Jordan University of Science and Technology, P.O. Box 3030, Irbid 22110, Jordan; rmdaghmash19@ph.just.edu.jo

**Keywords:** three-dimensional (3D) printing, transdermal drug delivery, 3D printing methods, microneedle types, applications

## Abstract

Microneedles (MNs) are considered to be a novel smart injection system that causes significantly low skin invasion upon puncturing, due to the micron-sized dimensions that pierce into the skin painlessly. This allows transdermal delivery of numerous therapeutic molecules, such as insulin and vaccines. The fabrication of MNs is carried out through conventional old methods such as molding, as well as through newer and more sophisticated technologies, such as three-dimensional (3D) printing, which is considered to be a superior, more accurate, and more time- and production-efficient method than conventional methods. Three-dimensional printing is becoming an innovative method that is used in education through building intricate models, as well as being employed in the synthesis of fabrics, medical devices, medical implants, and orthoses/prostheses. Moreover, it has revolutionary applications in the pharmaceutical, cosmeceutical, and medical fields. Having the capacity to design patient-tailored devices according to their dimensions, along with specified dosage forms, has allowed 3D printing to stand out in the medical field. The different techniques of 3D printing allow for the production of many types of needles with different materials, such as hollow MNs and solid MNs. This review covers the benefits and drawbacks of 3D printing, methods used in 3D printing, types of 3D-printed MNs, characterization of 3D-printed MNs, general applications of 3D printing, and transdermal delivery using 3D-printed MNs.

## 1. Introduction

Transdermal drug delivery (TDD) systems cover a broad array of non- and minimally invasive approaches for delivering drugs and vaccines via the skin without the use of injections [1]. The major advantages of TDD systems include easy drug penetration through the skin (which enhances patient compliance), bypassing the gastrointestinal tract metabolism and pH effects, and the possibility of obtaining a consistent concentration of the active pharmaceutical ingredient (API). TDD systems are now widely used in the delivery of vaccines and macromolecules, including influenza vaccines, parathyroid hormone, and insulin [2]. Additionally, TDD systems are used in the treatment of cardiovascular diseases, anxiety, Alzheimer’s disease, Parkinson’s disease, etc. [3].

Microneedles (MNs) are micron-scale needles [4,5] that can be composed of metals, polymers, silicone, rubber, and/or ceramics, which are designed for both epidermal (i.e., directly delivering into the cytoplasm and nuclei of epidermis cells) and intradermal (i.e., delivering into the dermis, or underneath the epidermis) delivery systems [6,7]. Polymeric MNs have received a lot of attention due to their cell targeting efficiency by using polymeric carriers, as well as their lack of toxicity [8,9,10,11,12,13].

MNs’ design can control the drug release. If a bolus release is required, then the drug substance should be integrated into a dissolving MN directly, and the rate of the release in this case is largely dependent on the MN’s dissolution rate. However, since the quantity of the drug is small, this restricts the dose to less than 1 mg, in an MN patch of a few hundred MNs. In case of sustained release, a higher quantity of the drug is needed; thus, the additional amount is added into a backing layer of the dissolving MNs’ array patch [5].

Transdermal MNs have permitted the transport of several active compounds across the skin over the last 20 years. Gerstel and Martin had the first MN patent in 1976, as MNs were presented without pain for a TDD system and prevented tissue damage. In 2004, Mark Prausnitz proposed that MN arrays could increase the transport of both small molecules, macromolecules, and supramolecular complexes. There have been significant advances in MNs’ applications. The phrase “microneedle” (MN) was originally used in 1921 [14] to describe a method of micron-scale slicing of echinoderm eggs. In 1971, the idea of MNs as a drug transport system was published, which included both hollow and solid MNs [15]. In 1975, the first drug-coated MN was produced [16]. In 1998, MNs were first used for in vivo studies [17], and then in 2001 for the delivery of genetic material [18], in 2002 for the delivery of vaccines [19], and in 2003 for the delivery of nanoparticles [20]. In 2005, dissolvable MNs were developed [21,22]. In the same year, hollow MNs were used for the extraction of samples [23,24], for cosmetic applications [25], and for diagnostic applications [22].

MNs are available in a variety of sizes (height; diameter at the base and at the tip). The proper size and design for MNs allows them to pierce the skin’s outermost layer to reach the layers underneath. This allows the delivery of loaded substances. These advantages, along with the ability of usage without professional help, make MN approaches patient-friendly [26,27].

MNs can be fabricated by several conventional methods, such as micromilling process, which includes wet and/or dry cutting [28]; photolithography, which can be combined with thermal- and photopolymerization [29]; wet and dry etching [30]; cleanroom-free molding [31]; molding-based techniques [32]; laser patterning [33]; injection molding [34]; drawing lithography [35]; and photolithography with an elasto-capillarity-driven self-assembly mechanism [36]. Many of these methods have limitations, such as being labor-intensive, requiring manual steps, and having high costs [37]. Thus, cost-effective and more accessible technologies are needed to produce MNs.

The International Organization for Standardization defines “3D printing” as “the fabrication of things by the deposition of a substance utilizing a print head, nozzle, or another printer technology” [38]. Thus, it is considered to be an additive manufacturing method that generates three-dimensional structures (i.e., solids or semi-solids), prototypes, and designs with consecutive films translating a 3D computer-aided design (CAD) model input to a tangible object. The 3D printing method is divided into four stages: vat polymerization, material extrusion, material and binder jetting, and powder bed fusion [34].

Recently, a broad variety of 3D printing technologies has opened up an exciting avenue of study to fabricate MNs [39,40] and allowed 3D printing to be used in numerous industrial fields [41,42], including fashion, aeronautics, pharmaceuticals, and medical devices—particularly dental and orthopedic devices [43,44]. 3D printing was first introduced in 1980 and started to grow progressively and quickly in the market until it reached over USD 9.9 billion, and by the year 2024 it is anticipated to reach USD 34.8 billion [45].

There are numerous reviews that have been published about MNs and 3D-printed MNs [34,46,47,48,49]. However, this review focuses mainly on the most recent developments in 3D-printed MNs, and on the application of 3D-printed MNs in TDD. It covers the various 3D printing techniques used in the manufacture of MNs, the advantages and disadvantages, and the materials used. It also covers the other applications of 3D printing in the pharmaceutical and medical industries, as well as the techniques used to evaluate MNs. Some examples from the literature on MN arrays that were produced using 3D printing technology are mentioned. Finally, the prospects of 3D-printed MNs are delineated in order to advance their application in various fields.

## 2. Benefits and Drawbacks of 3D Printing

3D printing is mostly well known for the capability of manufacturing patient-specific tailored products and the easiness of developing prototypes. Although the printers are expensive, 3D printing has an overall low production cost, which compensates for the high price of the printers. Additionally, 3D printing has no storage cost, and it is biocompatible, cost-effective, and has high output volume and high production rates. Extremely important benefits of 3D printing include improving the accuracy, efficacy, convenience, and safety of medicines or medical devices [34,41,42,50,51]. The use of 3D printing methods enables the fabrication of micro- and nano-sized objects [52], as well as biomaterials [53], in addition to the production of multiple devices that can be manipulated in the laboratory and in medical care applications [54,55,56,57,58].

However, there are drawbacks to 3D printing, which include restrictions on the raw materials used in printing. Additionally, the dimensions of the printed objects are limited, and problems related to patency are present, such as the existence of many patented techniques and methods, which raises the costs of the materials and methods, such as in stereolithography (SLA) and selective laser sintering (SLS). Furthermore, there is a lack of checking of the parallel manufacturing of hazardous materials [51].

## 3. General Methods for 3D Printing

**Figure 1:**
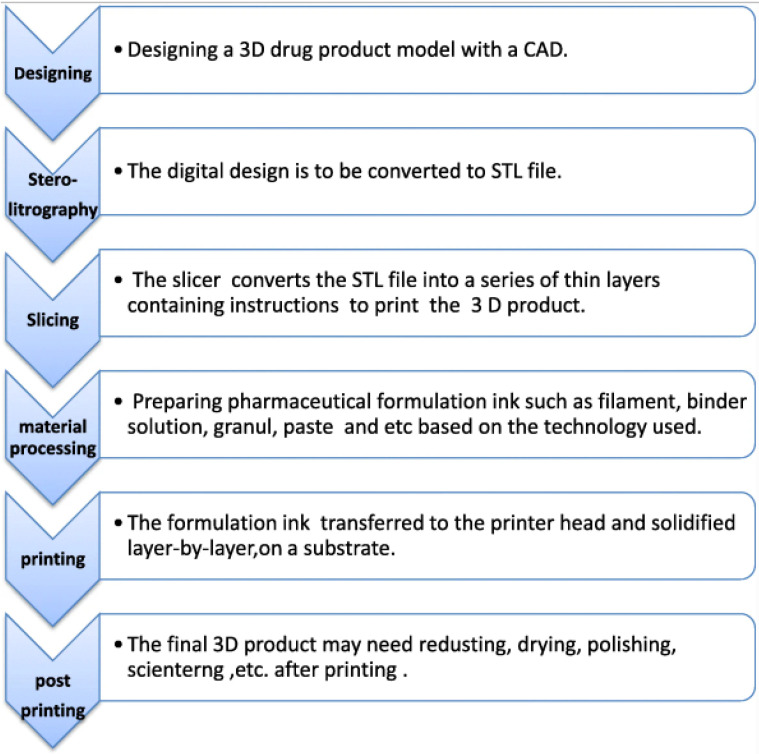
Multiple steps included in the manufacturing process of 3D printing [59].

3D printing begins with creating (virtual) models of the objects that are going to be printed. The design is generated in a CAD file utilizing a 3D modelling tool or a 3D scanner. Three-dimensional designs are often translated to the Standard Triangulation Language (STL) file format, which represents a 3D model’s exterior surface. Then, the 3D printing software will cut the exteriors into discrete printable layers and send the layer-by-layer digital instructions to the printer. The produced items may undergo further dehydration, annealing, polishing, or other post-processing procedures after printing [51]. The different steps are represented in Figure 1.

The American Society for Testing and Materials has classified the 3D printing procedure—or additive fabrication—into seven types, with reference to the basic terms of additive fabrication techniques. The following are the seven main techniques.

### 3.1. Binder Jetting

**Figure 2:**
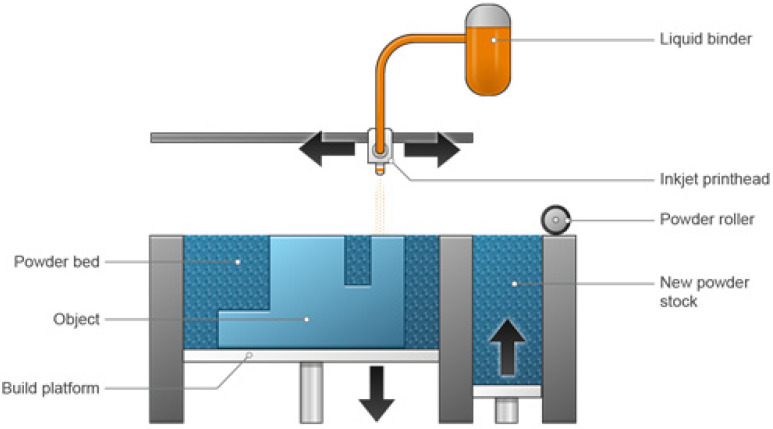
Binder jet 3D printing device [64].

In this method, inkjet printers spray the prepared formula of a pharmaceutical dosage form or binders (i.e., an active ingredient or an excipient) in the form of small droplets at a specified speed and size into a powder bed (Figure 2). The active ingredient can be injected as a powder, solutions, or nanoparticulate suspensions. This is the chief method used in the pharmaceutical industry, due to its accuracy and the ability to form accurate and precise objects [60]. Three-dimensional inkjet printing comprises three main steps: (1) drop creation, (2) drop influence and dispersal, and (3) drying or solidification. Most printing techniques conducted for the preparation of drugs exploit piezoelectric actuation, which involves the exploitation of great vapor pressure. The development of a droplet is not an easy procedure, and it is affected by the viscosity and density of the liquid, as well as the surface tension, among other factors [61].

There are many types of printers that employ the same technology but differ in the materials and binders used. Sands, ceramics, and metals are among the most typical materials used. However, plastics can also be used. Binder jetting can create parts that have acceptable tolerances, but it can be challenging to predict the final tolerance, because shrinking happens during post-processing [62].

Metal components are incredibly fragile before infiltration and can crumble if not handled carefully. They are nearly fully dense after infiltration, but their mechanical characteristics fall short of those of parts that are made in an old-fashioned way. These metal parts have smoother surfaces than those made with direct metal laser sintering (DMLS) and selective laser melting (SLM). Binder-jetted metal components can still be useful even though their mechanical characteristics do not match the strength or tolerances of powder bed fusion (PBF) prints when they are infiltrated and sintered [62].

This method is even more affordable than vat photopolymerization and PBF. For low-volume runs, print speeds are comparable to PBF and in line with other technologies, but they increase quickly as the volume increases. This method is perfect for full-color prototyping, because it can quickly and cheaply produce complex parts in a variety of colors. Binder jetting is less expensive than material jetting, and despite its limitations in terms of mechanical properties, it can still produce resolutions that are good enough for most prototypes. Binder jetting is particularly appealing for creating intricate sand casts because it can print large, intricate geometries at a reasonable price. Additionally, the procedure is easy enough to incorporate with the majority of conventional foundry processes [62,63].

### 3.2. Material Jetting

**Figure 3:**
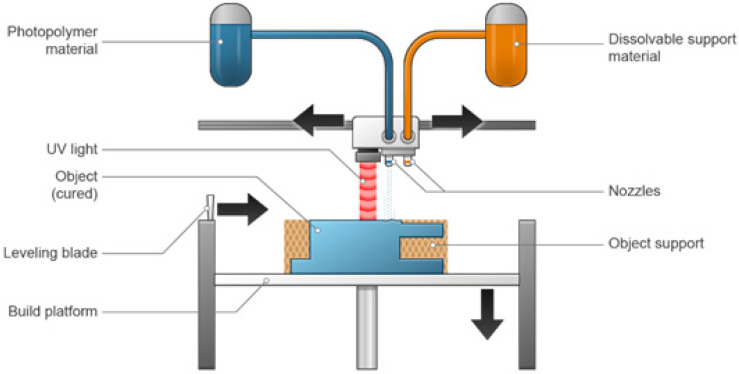
Material jet 3D printing device [64].

This is a different method from the previously mentioned method (binder jetting), in that it is more difficult to apply. The material jet device is presented in Figure 3. However, the benefit of material jetting over binder jetting is the ability to perform with high resolution. The inkjet droplets are as small as 100 μm in diameter and result in extremely thin layers. The jetted substances are liquefied polymers and waxes, resins, solutions, suspensions, and complicated liquids composed of numerous materials [40]. A wide variety of materials can be used with material jetting printers because of the wide range of technologies in the category. Photopolymers, metals, casting wax, flexible plastics, and ceramics are the most widely used materials. Full-color prints in a variety of materials and textures can be produced using PolyJet printers [62,63].

Despite the durability of the parts produced by material jetting printers typically being less than what fused deposition modelling (FDM) or PBF can achieve, they are extremely accurate and capable of producing parts with extremely high tolerances. Although the surface finishes are very smooth, printing in a matte setting is an option.

The cost of material jetting varies depending on the type of printer. There is a lot of material waste per part because the materials are expensive and the support structures are printed solid. Its speed is comparable to the production speeds of PBF. Although material jetting is an expensive 3D printing technology, it is the only practical option when dimensional accuracy or visually appealing designs are essential, due to its quite high dimensional accuracy and smooth surface finishes. Highly realistic prototypes, anatomical models, intricate and highly precise tooling, jewelry, medical equipment, and surgical tools frequently fall under this category. Prototypes for haptic feedback are frequently created using multi-material printing, such as a rigid case with flexible buttons [62,63].

### 3.3. Material Extrusion

**Figure 4:**
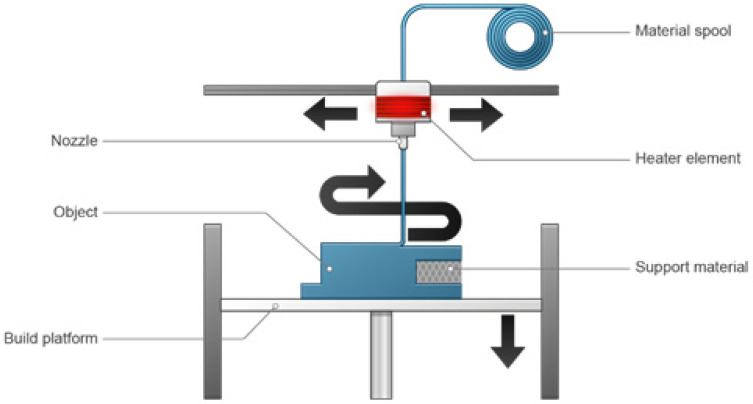
Material extrusion 3D printing device [64].

The substance is injected through a nozzle that is controlled by a robot. This method does not need a powder bed, and the printing can be carried out on any material. The material extrusion printing device is presented in Figure 4. Fused filament fabrication (FFF) or FDM is a type of extrusion printing that employs thermoplastic polymers such as polylactic acid, polyvinyl alcohol, and acrylonitrile butadiene styrene [65].

Although there are occasional exceptions, FDM printers are typically not used to create functional end-use parts. Since the parts that they print are weak along the z-axis, they are not among the most precise 3D printers. On all surfaces of the parts, layering is also obvious.

Industrial-grade machines can be considerably more costly and beyond the price range of even the most devoted hobbyists. Even though material extrusion machines are inexpensive to buy and simple to operate, outsourcing is still common because one-day lead times are now standard due to the technology’s widespread use. Standard FDM materials are widely accessible, and pricing is kept low by competition. Unlike vat photopolymerization or PBF, single-part printing is quick, but there are no scale-up advantages. As a result, FDM is comparatively slow for high-volume runs and probably not the best option for many different parts [62,63].

FDM parts are very affordable even though they are not as strong or aesthetically pleasing as parts created in other ways. Due to these qualities, FDM is the most frequently used prototyping technology, particularly at the proof-of-concept stage. If the resolution and surface finish are not crucial considerations, FDM’s availability of a wide range of materials, speed, and affordability make it highly desirable for certain types of production parts. Industrial FDM printers can quickly create working prototypes and finished products out of durable materials such as grips, jigs, and fixtures. FDM manufacturing is more cost-effective than traditional manufacturing when producing these latter components [62,63].

### 3.4. Powder Bed Fusion

**Figure 5:**
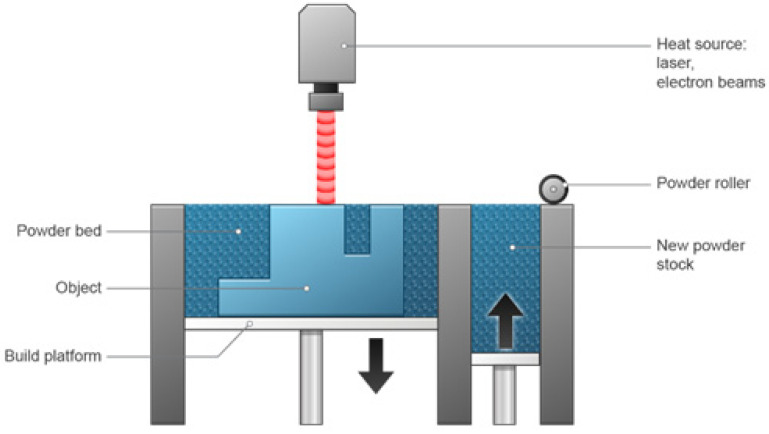
Powder bed fusion 3D printing device [64].

PBF includes sintering, which means fractional superficial liquefaction and congealing or binding of particulates that have high a melting point with a low-melting-point binder [65]. Printers create parts by sintering or selectively melting powdered particles to create an entire object. A thin layer of the powder material is applied to the build platform after being heated to just below its melting point. To create a single cross-section of the print, a laser or electron beam is then focused across the powder’s surface. The build platform descends, and the procedure is repeated after each layer. Up until all of the layers have been combined into one object, each new layer is fused to the one before it. The detached particles serve as a support structure for the print as layers are added on top of one another, obviating the need for most distinct support structures. The extra supporting powder is removed and recycled after the print is finished [63]. The device is presented in Figure 5. It is characterized by being a faster method than material extrusion; however, it is more complicated due to the need for a powder bed to be fused using a laser beam. Furthermore, it is suitable for substances that withstand heat, such as polylactic acid [66].

PBF technology is utilized by many 3D printers. SLS, DMLS, SLM, HP’s multi-jet fusion (MJF), high-speed sintering (HSS), and electron-beam melting (EBM) are the most widely used types of printers. For plastics, SLS is the most popular, while for metals DMLS and SLM are the most popular. These printers are also capable of printing high-resolution parts. PBF can create parts from extremely complex digital models, because unused powder serves as a support material as the print layers are built up. Both MJF and SLS have a similar potential for complexity, both outperforming SLA. Although SLS always prints at 100 microns and MJF at 80 microns, SLA has even higher resolution (its layer height can go as low as 25 microns). SLS has a wider selection of materials, but MJF can produce slightly better resolutions. DMLS, SLM, and EBM are able to print parts for metal with some of the greatest resolutions currently available [63].

PBF can produce tolerances that are comparable to those produced by vat photopolymerization, but PBF parts are much more robust. PBF is able to create plastic components that are both functional and have the best mechanical qualities of any 3D printing technology. MJF prints have a smoother surface finish and are marginally stronger than SLS prints [62,63].

EBM systems have a lower potential for distortion because they generate fewer residual stresses than DMLS and SLM. Since the powder particles are only lightly sintered and the parts are still slightly porous, the metal parts produced by DMLS are not as strong as those produced by SLM. SLM parts, however, can match traditional manufacturing processes such as forging and machining in terms of their mechanical properties. Due to the use of powders in their creation, PBF prints all have a slightly rough finish, but they are easily polishable.

Although PBF market competition keeps prices down, it is still expensive. The cost of 3D printing metal is still significantly higher than that of computer numerical control (CNC) machining. The price is comparable to that of vat photopolymerization for plastics. Typically, MJF is about 10% less expensive than SLS. SLS and MJF take longer to produce low volumes of plastics than vat photopolymerization and FDM. However, because components are printed directly on the build platform, they are the quickest for large quantities. PBF is the favored method for producing low volumes of plastic parts across all industries, because it can create strong functional parts. One-off industrial hardware—such as machine parts, jigs, grips, and fixtures—and low-volume production runs of specialized plastic parts are typical applications. PBF is the preferred technology for rapid prototyping because it can make extremely complex parts [62,63].

### 3.5. Photopolymerization

**Figure 6:**
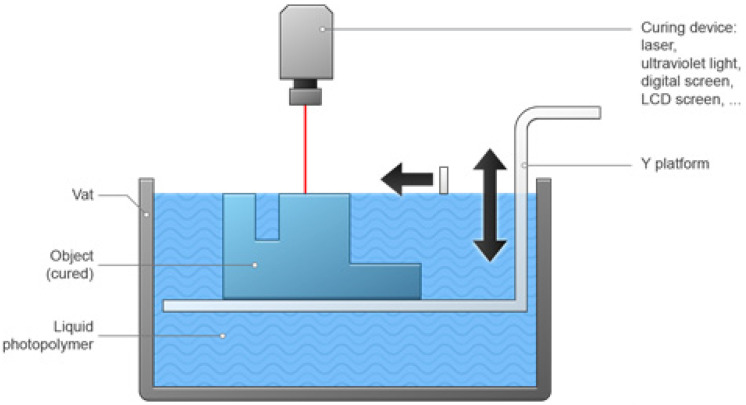
Photopolymerization 3D printing device [64].

This method is also known as SLA [66]. A polymerization reaction is initiated when a liquefied resin is passed through ultraviolet (UV) or any other high-energy light, as shown in Figure 6. A photopolymerizable raw material is needed for this method. Photopolymerizable hydrogels are examples of materials printed with this technique [67].

SLA, direct light processing (DLP), and continuous liquid interface production (CLIP) are the three most popular subtypes. They are very similar in terms of how a light source directs light at the resin. Overall, SLA is the vat photopolymerization printer technology that is most popular and widely used.

Photopolymer resins, the majority of which are proprietary, are used in this method. Standard resins for all-purpose prototyping are some of the many types that are readily available. In addition to these, there are also strong acrylonitrile-butadiene-styrene-like resins, elastic rubber-like resins, transparent castable resins with no ash content after burnout, ceramic-filled resins for extremely rigid prints, and biocompatible resins for medical devices [62,63].

Vat photopolymerization is capable of printing extremely complex parts, but they cannot produce parts as complex as PBF because they require support structures. These devices can print extremely fine details [62,63].

### 3.6. Directed Energy Deposition

Directed energy disposition (DED) uses a direct energy source such as a laser or electron beam to melt down raw substances, after which they are deposited. The DED process involves layering beads of molten material—typically metal. The method of printing is essentially the same as that used in metal extrusion printers for plastic. The feedstock material, which can be wire or a powdered form, is continually pushed via a nozzle and melted at the point of deposition by a laser, electron beam, or arc before cooling and solidifying [63].

**Figure 7:**
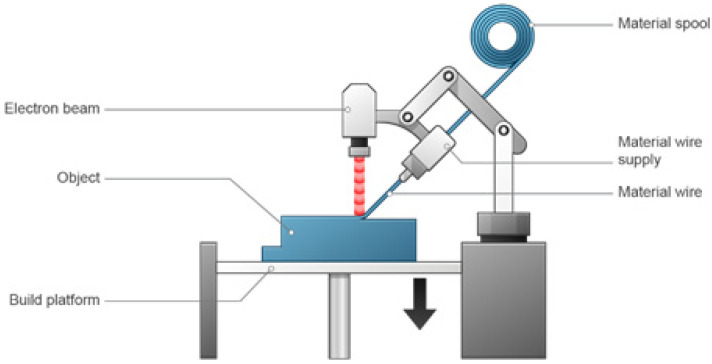
Directed energy deposition 3D printing device [64].

The DED 3D printing device is presented in Figure 7. This procedure is used for powders and substances that spoil upon extrusion [66,68]. Both metals and ceramics can be printed using this technology, but ceramics are far more frequently used [63].

The enormous print-bed sizes of DED are yet another significant benefit. Large manufacturers frequently construct DED printers for customers with build envelopes that are several meters long along any dimension. Because there is no room for overhangs due to the size of the liquid melt pool at the deposition point, support structures are possible but challenging. The same characteristic makes complex geometries impossible. Compared to other metal 3D printers, the resolution is very poor. Powder particle sizes range from 50 to 150 microns, and the diameter of the welding wire is 1 to 3 mm. For instance, sharp corners can only be produced through post-processing [63]. Although DED generates completely dense parts with mechanical characteristics that are equivalent to those of metal parts, the tremendous amount of energy needed to keep a melting point at the point of deposition develops large thermal gradients that may result in a lot of residual stress. However, because of the low resolution, the parts frequently have a poor surface finish, necessitating secondary machining to produce the best results [63]. The printing speed is high (very low resolution), and the material cost is less expensive [62,63].

Part repairs, feature additions, and near-net-shape part production are the three primary uses of DED. This makes it perfect for adding features and fixing damaged parts that cannot be added using other processes. The vast majority of uses involve tool repair, and businesses turn to DED when expensive machinery can be repaired more affordably than it can be replaced, such as in heavy industry [62,63].

### 3.7. Sheet Lamination, Automated Laser Cutting, and Sheet-By-Sheet Assembly of Products

By stacking and laminating sheets of a substance with single horizontal cross-sections, sheet lamination creates parts. Several printers laminate the sheets after they have been cut. In the majority of cases, the sheets are laid, laminated, and then cut to size [63].

This is among the simplest ways to construct 3D models. Even though it is straightforward, there are numerous distinct proprietary technologies based on the material, lamination technique, and cutting technique. Most of the time, the procedure is a straightforward variation of laminating objects from paper. The only radically different technology is ultrasonic consolidation, which employs ultrasonic welding rather than a different bonding substance.

There are many materials that can be used with printers of all kinds, including papers, most polymers, fiber-reinforced polymers, ceramics, and almost any metal. All of these materials can be used to create multi-material layers as long as they can all be laminated and shaped using the same techniques [63].

Although the size of sheet lamination print beds varies considerably, they are comparable to those of SLA and SLS printers. The use of large-format printers is uncommon. Highly complex shapes are not possible, because the sheet-cutting techniques are so basic. However, internal structures are possible, because support structures are not required. Embedded wiring between sheets is an additional design choice. Since most processes do not need heat, there is less of a chance that high temperatures will ruin them. The material feedstock is the only factor that influences the resolution. However, because of the brittleness of the bonds between the sheets, these parts cannot be used for structural or functional uses [62,63].

This technique is cheap and fast, but it has low accuracy and wastes a lot of raw materials [66]. This method was initially applied for the construction of models in architecture. Today, proof-of-concept and look-and-feel prototyping are typically used for highly detailed, colored objects [62,63].

Numerous methods are available for 3D printing; however, only a few are used in the medical field due to the need to use materials that are pure and compatible with the body. These techniques are powder-based printing [69], vat-polymerization-based printing [70], droplet-based printing [71], and extrusion-based printing [72].

## 4. Fabrication of MNs

The manufacturing of 3D-printed MNs comprises three key stages:

(1) A scheme of the requested design is obtained digitally using the CAD software, and the dimensions are improved using printer specifications.
(2) The scheme is transformed into a Standard Tessellation Language format, transferred to a computer that manages the 3D printer, and the size and orientation of the printing are selected [73,74].
(3) The item is printed in the form of continuous layers [75].

3D printing techniques are categorized according to the source of energy, types of substances used, or any other mechanical properties. Three technologies are widely used in the pharmaceutical industry, which are discussed in the following subsections [40,76].

### 4.1. Nozzle-Based Deposition Systems (Fused Deposition Modelling)

This approach is also known as extrusion-based FFF, or FDM. It is a common technology in the fabrication of MNs. It is an easy and uncomplicated method that is used for plastic materials that are melted as a filament and liquefied using a liquefier head. The temperature used is higher than the melting point of the plastic material. Afterwards, consecutive layers of the melted plastic are deposited on a plate and through a nozzle. The layers cool and solidify in a rapid manner. The x- and y-axes control the movement of the printer’s head, while the z-axis controls the platform; thus, a 3D object is created [77].

Many features control the quality of the produced MNs, including the process parameters—for instance, the selection of the nozzle diameter and plate diameter, the feeding rate, the speed of the printer, the thickness of the layers, and the positioning of the built object. Therefore, it is important to study these parameters thoroughly in order to reach the best and optimized specifications, giving a final product that has a good surface finishing and is strong and resistant to mechanical stresses, in addition to other properties that are required in the printed object [78,79]. The filament dimensions that are used in the FDM print head are between 1.75 mm and 2.85–3 mm, and the crucial feature is thermoplasticity [73]. This method is reasonably priced, highly reliable, fast, and uses cheap substances. It has many disadvantages in comparison with other 3D printing processes, such as having a slight resolution [77,80] and innate restrictions in terms of dimensional precision and surface texture [81].

Many types of MN arrays have been printed with polylactic acid. They have the advantages of being recycled, biodegradable, FDA-approved, thermoplastic materials. When the final steps of manufacturing were followed by chemical etching using an alkaline solution, MNs with sharp tips were produced, without affecting the other mechanical and chemical features [80]. To solve the accuracy issue of the FDM method, researchers have discovered that thinner layers lead to more accurate tips [81].

FDM printing has been used to produce hollow MNs, and through tailored programs the release profiles of various drugs were controlled—for example, in the case of vascular endothelial growth factor, which helps in wound healing, hair growth, and angiogenesis [82].

### 4.2. Laser-Based Writing Systems

#### 4.2.1. Stereolithography

The photopolymerization-based or photocuring 3D printing technique is among the first invented methods and is a very common technique. It uses laser emissions or light projections in order to polymerize photosensitive polymers [83]. Vat photopolymerization methods such as SLA, which are liquid-based procedures, have the advantages of good precision and accuracy. SLA is based on the principles of a highly organized layer-by-layer solidification of a photosensitive liquefied resin upon scanning with a laser beam [83]. SLA printing machines consist of a printing platform, a resin tank, and a UV laser that outlines a transection on the polymer resin, causing the transection to harden [84].

Based on the filling method, the SLA procedures can be classified into two major groups:

(1) Free surface: objects are created from the bottom up in a support platform that resides directly beneath resin surface.
(2) Constrained surface: represented as “bat” configuration, where the platform has a building platform that is suspended over a resin bath [83].

The SLA method is a very common printing technology due to the reported features of being able to manufacture solid MNs that are smaller than 100 µm, with outstanding mechanical strength and penetration capability [11].

Yao et al. (2019) have proven that precision and stiffness can be affected by the exposure time of each layer through creating a high-precision digital light processing (H-P DLP) 3D printing that is based on light curing [85]. The same result was concluded through testing various shapes of MNs that were manufactured by the invented system, which were made with biocompatible substances and various printing parameters [86].

#### 4.2.2. Digital Light Processing

DLP depends on photopolymerization-based technology. It is similar to the SLA method, but the only difference is the light source. DLP has more speed than SLA, using a smart projector that increases resolution, such as a digital micromirror device (DMD) [37]. It has a great printing resolution that can reach a minimum size of 50 µm. The resulting printed items have even and highly accurate surfaces. However, DLP 3D printers are extremely costly [75].

Hollow MN arrays were made from photolabile acrylate-based polymer resin using DMD™-based micro-SLA [87]. Solid MN arrays with diverse geometries could be prepared using acrylate-based polymers through DMD™-based stereolithography. For preservation and providing antimicrobial results, coatings could be obtained through the pulsed laser deposition technique and using the materials silver and zinc oxide [88].

#### 4.2.3. Liquid-Crystal Display

Another type of vat polymerization technique is known as liquid-crystal display (LCD). The main approach uses a resin that solidifies the layers using UV light. In LCD 3DP, the fluid crystal is employed as a photo-taking structure. The advantages of the bottom-up 3D printing process over top-down techniques is that it achieves great upright resolution with less curing time and lesser quantities of resin for the fabrication process [75,89]. Accuracy and precision are higher in LCD than in DLP. LCD is used to manufacture hollow MNs [90].

#### 4.2.4. Continuous Liquid Interface Production

CLIP is one of the newest technologies in 3D printing, invented in 2015. This technique, in modern procedures, replaces the original layer-by-layer SLA [40]. The method’s main principle is dependent on the innovation of a membrane that allows for oxygen diffusion, which aids in the successive printing for the oxygen permeation and prevents radical polymerization [75]. The procedure starts when a beam of UV light is directed into a photopolymerizable liquid resin through an oxygen-permeable window, which is selectively polymerized through UV radiation. Above the window, a liquid “dead zone” of non-polymerized oxygen-inhibited resin is maintained, which allows constant (rather than layer-by-layer) construction of the object. The advantages of CLIP the technique are its high speed of printing and high resolution. However, this method is expensive and is not yet readily available or convenient [40,91].

#### 4.2.5. Two-Photon Polymerization

Two-photon polymerization (TPP/2PP) is considered to be the most accurate 3D printing technology. It allows for the printing of consecutive layers of objects from a different variety of substances—either fluids or solids—at the micro- or nanoscale. The main energy source is a near-infrared femtosecond laser. TPP has the highest resolution, with the ability to reach up to 100 nm horizontal resolution and 300 nm axial resolution [92]. The method depends on two-photon absorption. As a procedure, it has many benefits over traditional methods: it is a quick method requiring only one step, it can easily be converted into mass production (i.e., scaled up), and it does not require cleaning after implementation, in addition to the ability to utilize inexpensive substances (such as ceramics) and other materials, such as photosensitive materials and polymers [92,93,94]. 

Many types of MNs have been successfully fabricated by researchers. For instance, Moussi et al. (2021) succeeded in printing hollow MNs that could be embedded in the body using this procedure, along with an associated storage chamber [95]. Doraiswamy et al. (2006) used this method (TPP) to print MNs using ORMOCER^®^—a modified ceramic that is biologically safe and non-toxic—which showed important characteristics of mechanical durability when they punctured into the skin [96]. Ovsianikov et al. (2007) reported that hollow MNs manufactured using the TPP technique can have numerous structures, such as cylindrical, conical, and pyramidal [97,98].

#### 4.2.6. Powder Bed Technologies

This method was first described by Carl R. Deckard, and he obtained a patent for it in 1989 (US 4863538 patent) [99,100]. It depends on powders as the basic substances for 3D printing and on the usage of high-energy methods such as SLS, SLM, and direct laser metal sintering (DLMS). The powder flows on a bed for the structuring of the required items [101]. Sintering and melting are two methods employed to solidify the printed material [73]. This technique also follows the principle of consecutive layer printing based on a high-power laser beam that is automated using a computer system [101]. The powder material—e.g., metal, plastics, polymers, or ceramics—is heated and forms strong bonds upon hardening, with the required structure and features [101]. DMLS is considered to be a method that is closely related to the SLS 3D printing technique, in which a high-intensity laser beam is used for sintering the final product [102]. The powder bed used is packed with a metallic blend of powders, such as bronze or 316 L stainless steel, along with other materials, without the need for binders and a fluxing mediator [103].

The method begins with CAD data from the file formatted as STL and is followed by a uniform dispersion of the powder onto the structure’s platform with the help of a roller and a slot feeder [104]. The final finishing of the 3D printing is carried out through unsintered powder molecules that exist on the bed, and afterwards they are discarded manually or by using vacuuming or sieving [104]. Additionally, DMLS has the ability to manufacture microstructures with complicated shapes, with good resolution and accuracy [103]. Sun et al. compared three 3D printing procedures in the printing of models to simulate MNs:

- DMLS of 316 L stainless steel (SS).
- Lost-wax casting of sterling silver using DLP/SLA-printed wax masters.
- Binder inkjet printing of 316 L SS.

Binder inkjet printing of 316 L SS MNs showed very few in-plane and out-plane deviations. Binder inkjet printing was also able to adjust the final shape with high accuracy. However, DMLS synthesized tiny MNs with finishes that were less accurate compared to the binder inkjet printing [103].

### 4.3. Inkjet Printing

**Figure 8:**
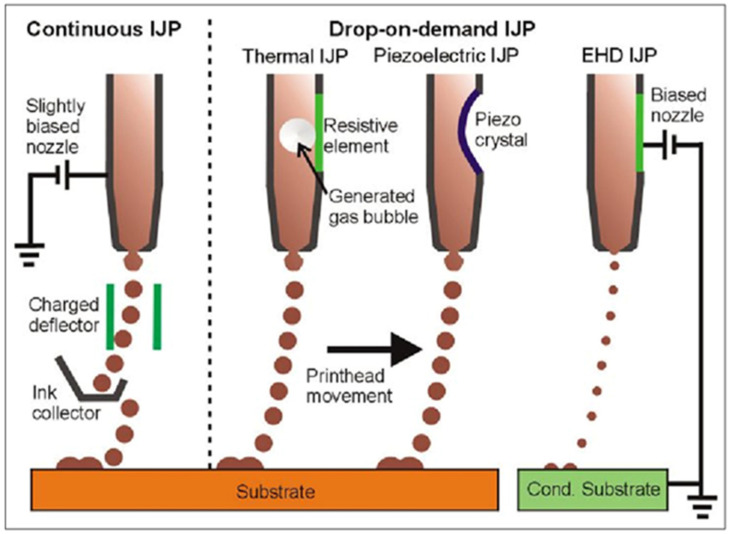
Schematic representation of CIJP printing and DOD IJP printing [107].

Inkjet printing is a method that is based on Lord Rayleigh’s instability theory, which clarifies the breaking of a liquid into jet droplets [105]. This principle is used to design a continuous jet (CJ) and drop-on-demand (DOD) printing. Tiny droplets are charged as they exit the nozzle and are directed by electrostatic plates to the substrate or to recycling [106]. CJ printing is represented in Figure 8. The advantage of the DOD method is that the actuation of the droplets is precise and less wasteful in terms of ink usage.

The benefits and drawbacks of various 3D printing methods, in addition to the materials used are summarized, in Table 1.

## 5. Transdermal Drug Delivery Using Microneedles

TDD systems include an extensive range of non-invasive or minimally invasive techniques for administering medications and vaccinations via the skin without the use of needles [1]. MNs can be useful in pain management [118]. One important application for TDD using MNs is the diagnosis of diseases through inserting hollow MNs, withdrawing interstitial fluid, and studying the contents [119]. Moreover, painless TDD can be important for cosmeceuticals [33,120].

MNs have the characteristic of causing less pain or even no pain due to their microscopic dimensions, in addition to being user-friendly [121]. Moreover, patients can take the needle at home, since it does not need professional staff, reducing the possibility of acquiring infections due to multiple hospital visits. Additionally, a smaller quantity of the antigen-producing cells present in a vaccine is required when using MNs as compared to traditional methods of vaccination. MNs also have the benefits of avoiding the storage of cold chains and having the flexibility of self-operation, which may overcome the logistical and delivery challenges of vaccines, enabling easier and more convenient immunization of the special population. Solid-coated MNs have dry coatings, making the MNs more stable and enabling them to be stored at room temperature. Nevertheless, there are some drawbacks regarding MNs: the limited quantity of drug that can be given via MNs means that they are only suitable for potent or low-quantity drugs; skin thickness differences due to age, race, and/or sex can cause variations in TDD; there are no available cost-effective studies comparing the different printing methods; and there is variation in cost depending on the ink and materials used.

## 6. General Medical Applications of 3D Printing

### 6.1. Tissue and Organ Models

Models are vital for studying diseases. The formation of such models using conventional techniques requires a large number of experimental animals. For instance, patient-derived xenograft models for pharmacological studies are costly and time-inefficient due to the need for a huge quantity of immunodeficient mice to engraft disease cells; thus, 3D printing can overcome this disadvantage through accurate, ideal, biomimetic models with high resolution in a time- and cost-saving matter. The organs include complicated structures such as the kidneys, skin, liver, and various tumor types. For example, 3D livers encapsulated in hybrid hydrogels were printed through vat polymerization technology [122]. Additionally, human intestinal cells and liver cells were 3D printed in order to study their ability to differentiate and remain viable. Moreover, the relationship between the two organs was studied [123]. Skin tissue was also printed with skin-derived extracellular matrix bio-ink and with endothelial progenitor cells and adipose-derived stem cells. This model was generated in order to study wound healing and neovascularization [124]. Tumor models were also fabricated through 3D printing along with the presence of tumorigenic influences, including the complicated microenvironment and microstructure. Three-dimensional printing enabled simulation of the tumor stroma and microenvironment with high accuracy, allowing for experiments with modern drugs [125].

### 6.2. Medical Apparatus and Instruments

Conventional methods have been used to manufacture medical implants and prostheses for hundreds of years, and over the years they have shown some issues, such as structural and functional mismatching, incomplete binding, lack of strength and primary steadiness, low bone development, long-term stability issues, and low cost-effectiveness [126]. These drawbacks have been overcome using 3D printing, enabling the manufacture of implants with accurate dimensions and functional characteristics.

**Figure 9:**
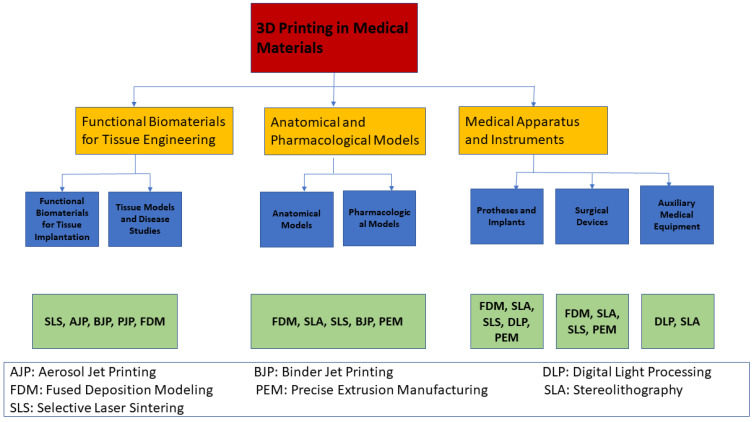
Progressive 3D printing technology and its application in medical materials. Chart showing the application area (yellow boxes), with corresponding products (blue boxes) and primary 3D printing techniques (green boxes) [45].

**Figure 10:**
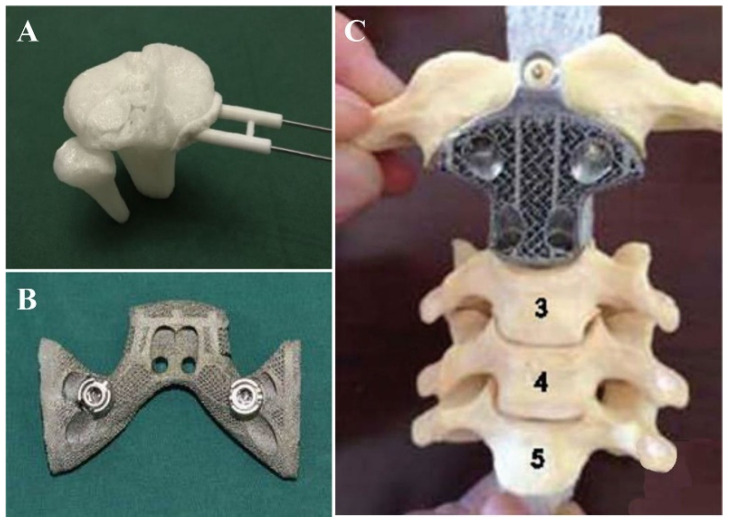
Medical devices manufactured through 3D printing techniques (**A**) Surgery simulation guide template. (**B**,**C**) Titanium apparatus for cervical spine and pelvic surgery respectively [45].

On the other hand, 3D printing offers great manufacturing prospects for medical devices (Figure 9 and Figure 10). It provides structurally fitted devices that are safe for both medical professionals and patients. Moreover, 3D printing can synthesize complicated micro-objects that cannot be produced by traditional methods. In addition to being a fast technique, it also has low waste of materials [127]. Powder-based 3D printing methods are used in the fabrication of implants, since the printing ink is biocompatible; examples of bio-inks used in 3D printing include titanium alloy, zinc alloy, cobalt–chrome alloy, and polyether ether ketone. Implants produced by 3D printing are being used in tracheobronchial, dentofacial, cardiovascular, orthopedic, and spinal surgeries [49,128]. Metallic tracheobronchial expandable stents, which are produced using the SLS 3D printing method, are being applied in patients with severe tracheobronchomalacia to prevent collapse of the bronchi and allow for the rebuilding of the airways [49,129,130,131,132,133,134,135,136]. In addition, 3D technology has enabled the fabrication of intricate facial constructions with data obtained from MRI or CT imaging. Imbalances in these structures make it extremely hard to obtain the desired shape of the implant using the conventional method (hand-curved wax model) [129]. Cardiovascular diseases have been broadly studied using 3D techniques—especially cardiac jetting, SLS, FDM, and SLA [132,133,134,136].

### 6.3. Three-Dimensional Printing in Tablets for Oral Drug Delivery

Powder bed printing was the first 3D printing method used for tablet fabrication [137,138]. 3D printing allowed for the introduction of very small amounts of drugs in the tablets and permitted physical characterization of the tablets, which exhibited good hardness and friability. The powder printing technique (3D inject printing) yields more permeable and porous tablets when compared with normal compression methods. This has led the utilization of 3D printing techniques in the manufacturing of highly soluble orodispersible tablets (ODTs) [139]. Three-dimensional printing methods have also been used for the production of modified-release tablets such as intermediate-release, extended-release, delayed-release and pulsatile-release tablets, multiple-API tablets, pediatric-printed tablets, and buccal films for oral delivery. For example, paracetamol extended-release tablets and levetiracetam ODTs were fabricated through powder bed injection [139], and modified-release tablets containing 5-acetylaminosalysilic acid were synthesized by the FDM procedure [140]. The complex geometry of these types of tablets is hard to attain through direct compression and requires more complicated procedures. However, 3D printing permits easier application to produce very thin layers and efficient barrier layers on the top and bottom; this structure reduces the surface area due to the outer part, and it also gives rise to an increase in the surface area of the inner part, leading to zero-order drug release [137,141,142].

### 6.4. Three-Dimensional (3D)-Printed Implants

Biodegradable implants have been developed for drug delivery [61]. Wu et al. (2009) created several systems using methylene blue as a colorant for active constituents, using polyethylene oxide as the polymer matrix and polycaprolactone as the rate-limiting constituent. These investigations demonstrated the capacity to place small droplets of dye solution accurately, as well as microstructures. This approach demonstrated the possibility for greater control over shape, surface area, spatial deposition, and other factors that impact drugs’ release kinetics. Thus, the release kinetics was affected by the spatial deposition, surface area, and other factors. These implants decreased or eliminated the burst effect and achieved more precise release (i.e., zero-order release) than implants manufactured by traditional processes such as injection molding or compression [61,143,144].

Three-dimensional printing is used for the production of implantable dosage forms. In 2007, Huang et al. (2007) created monolithic levofloxacin implants for comparison with compression implants and implants with complicated design for pulsed and bimodal release [145]. Because the 3D-printed implants were considered to a have more porous architecture than the compressed ones, it was found that the release of the drug from the printed ones with a small burst release was faster than that from the compressed ones [106]. Pulsed and bimodal drug release was shown using implants printed with an internal layer as storage for therapeutic agents, with another film of drug in the external layer. The implants were demonstrated to have burst- or pulse-release capacities of up to 400 mg and a steady-state capacity of 120 mg or less for up to 90 days [145]. The above group used the bimodal configuration of the implant to deliver levofloxacin and rifampicin. The delayed release of the internal storage chamber of rifampicin given on day 8, with sustained release of both APIs for 6 weeks, demonstrated the ability to prepare combinations with multi-mechanism release behaviors [146].

Devices and medical implants, such as stents and catheters, are often coated or spray-coated with materials for localized effects [42,147]; however, 3D printing enables enhanced efficiency, volume, and spatial control. Tarcha et al. (2007) demonstrated the capacity to formulate therapeutic solutions onto stents, employing CJ print heads for a low-dosage coating, on different stent geometries. This enhanced the precision and reproducibility, as well as the coating efficiency with typical coating methods [148].

## 7. Three-Dimensional (3D)-Printed Transdermal Delivery Systems

TDD systems include an extensive range of non-invasive or minimally invasive techniques for administering medications and vaccinations via the skin without the use of needles [1]. Transdermal delivery devices are useful for avoiding pH-mediated degradation and/or first-pass metabolism, as well as for making administration easier and without pain for people with diabetes and other chronic diseases. One important application for TDD using MNs is the diagnosis of diseases through inserting hollow MNs, and withdrawing interstitial fluid, and studying the contents [119]. Moreover, painless TDD can be important for cosmeceuticals [33,120], and it is useful in pain management [144].

## 8. Drug Delivery Using Microneedles

An array of solid MNs were inserted through the skin in an earlier stage of microneedle research to overcome the stratum corneum’s barrier effect. After treating the skin surface with silicon wafer-based needles, a medicated patch was placed on it. The interstitial fluid was also extracted using this method to measure the glucose level non-invasively [24].

The development of solid MNs coated with drug solution using a dip-coating method was the main goal of subsequent research in microneedle technology. In this ‘coat and poke’ method, only a small amount of drug (about 1 mg) could be coated on top of MN\s, necessitating extensive optimization for uniform coating.

A “poke and release” strategy was created as a result of additional research. The “poke and release” method had the advantage that the drug release could be controlled to meet the needs using a variety of readily available polymers and polysaccharides. In contrast to other physical approaches, the administration of a large dose of medication was still impractical with soluble or biodegradable microneedles, which prompted the creation of hollow microneedles. This method, called “poke and flow” involved permeating the skin and allowing the drug to flow through hollow microneedles from the reservoir in the patch afterward [149,150,151].

Hollow microneedle arrays with a drug reservoir were created by Wang et al. (2009) [152]. When the reservoir is put under pressure from the outside, the microneedle system enters the skin and the drug solution is then released into the skin. Thus, the creation of hollow microneedles allowed for the administration of large doses of medication. For ease of microneedle insertion and to avoid channel blockage, pores were typically kept adjacent to walls rather than in the center.

For effective drug delivery, the microneedle’s design must be such that it does not break and does not cause pain or irritation. Drugs can be delivered either systemically or locally using any of the methods mentioned above.

## 9. Materials Used in 3D Printing of MNs, and MN Types

The materials used in MN fabrication must be simple to manufacture, strong enough to penetrate the skin barriers, compatible with the active ingredient, biodegradable, and safe. The choice of materials will affect the tensile strength or hardness, loading efficiency, stability, and biocompatibility of the MNs [153,154]. Each type of MN has its own fabrication material, depending on the 3D printing procedure used; these will be discussed under each type.

MNs can be divided into hollow and solid in terms of their structure, and they can also be classified into five categories in terms of application.

### 9.1. Hollow MNs

These are manufactured using numerous substances, such as glass, polymers, metals, and ceramics [97,155,156,157]. Their diameters range from 5 to 70 µm, and they apply a poke-and-flow mechanism through passive or active diffusion (which can be obtained through pressure or a pump) [158,159]. Hollow MNs are used for the administration of a constant flow of a substance or a drug, such as insulin [155,160]. When using an MN array supported by a micropump or microfluidic chip [161,162], this permits storage of the drug. Blockage of the MN tip can occur upon administration of the drug, and this is a drawback of hollow MNs [121]. This can be resolved by adding eccentric holes [158], or by adding hyaluronidase to the mixture to increase the infusion rates up to 300 mL/min by reducing the infusion pressure and the skin flow resistance through the breakdown of hyaluronan—a glycosaminoglycan. Another solution to this problem is to moderately retract the MNs when injected to allow for the relaxation of the flattened skin over the MN tips [121].

### 9.2. Solid MNs

Solid MNs are composed of stainless steel [163,164], silicon [162], nickel [165], titanium [69], or polymers [161]. They can penetrate deeper into the skin to permit faster diffusion [166]. A poke-and-patch mechanism controls the drug release in solid MNs [167]. These MNs penetrate the subcutaneous (SC) tissue to generate momentary aqueous microchannels before applying a patch filled with the required substance or drug. The dosage form used can vary, including a wide range of lotions, ointments, gels, creams, foams, solutions, and sprays [159,168]. The substance in the batch is then released and diffuses passively through microchannels into the circulation [121,159], greatly enhancing the permeability [17,159]. In comparison with hollow MNs, solid MNs have greater resistance to mechanical stresses and they are easier to fabricate [166]. However, the dosage form cannot be controlled accurately [169]. The “poke and patch” methodology is also defined as the “scrape and patch” technique, since scratching of the skin is first performed by the microblades of the MNs, and then the drug is released through a patch and absorbed into the skin through microabrasions [167].

### 9.3. Coated MNs 

In this approach, the treatment formula is coated onto the solid MNs, and the drug formula dissolves into the punctured skin [170]. This type of MN and approach are considered for a single dose and used for an extensive variety of hydrophilic or hydrophobic therapeutic agents, including nucleic acids [171], proteins [172], and peptides [173,174]. The limitations to the coat-and-poke approach are the insufficient capacity of the MNs and the limited reservoir in both the base and the shaft [121,170]. This makes this type of MN only applicable for potent drugs such as vaccines, especially in the case of dense coatings. Owing to the inadequate sharpness of the MNs, this results in weak drug permeation into the skin [121,175,176].

### 9.4. Dissolving MNs

Dissolving MNs can be composed of several substances, such as polyvinylpyrrolidone, polyvinyl alcohol, carboxymethylcellulose, chondroitin sulfate, and sugars such as maltose, dextran, or galactose [177]. The release kinetics of the drug relies on the extent of the polymers dissolved; therefore, controlling the polymeric composition of the MNs can modify the drug release, in addition to adjusting the fabrication technique [121]. Since hydrophilic materials are utilized in the fabrication of MNs, this leads to a low possibility of hazardous biological residual solvents and, therefore, is considered to be environmentally friendly [121]. These MNs use the approach of “poke and release” [14]. They comprise a soluble matrix that includes biodegradable substances such as biodegradable polymers and sugars, or that contains the treatment agent within the matrix [121]. Biodegradable MNs dissolve once they come into contact with the interstitial fluid; afterwards, the drug is released [121].

### 9.5. Swelling MNs or Hydrogel MNs

**Figure 11:**
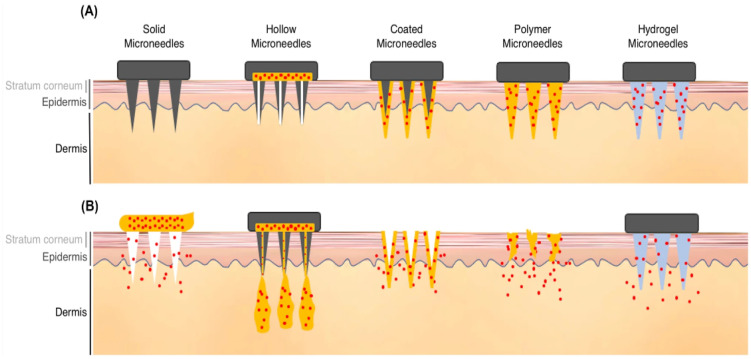
Different types of microneedles and their characteristics. (**A**) The structures of solid, hollow, coated, polymer, and hydrogel microneedles. (**B**) Each of these microneedles has different drug delivery properties. Solid microneedles are well suited for penetration and increasing drug permeability. Hollow microneedles create pathways for drug infusion. Coated microneedles contain drugs on their surface that dissolve after insertion into the skin. Microneedles made with biocompatible and biodegradable polymers contain drugs that fully dissolve in the skin to release their encapsulated reagents. Hydrogel microneedles made with non-dissolving, liquid-absorbing materials can be used for fluid and materials diffusion [180].

Swelling or hydrogel MNs are composed of aqueous blends of polymeric substances such as poly(methyl vinyl ether-alt-maleic anhydride) [121]. They contain a hydrogel-forming matrix [178], which can be used for both absorption of interstitial fluids and TDD. The drug is merged with a crosslinked polymer microprotrusion. When applied to the skin, the MNs swell and extract the interstitial fluid; consequently, this causes drug dispersion through the swollen MNs [121]. Hydrogel MNs can be used for monitoring analytes in bodily fluids, since swelling occurs once they pierce the skin and collect interstitial fluid [179]. These MNs can then be analyzed to obtain data on the existing analytes or biomarkers in the plasma. Thus, they allow observation of illnesses without causing pain to the patient [164]. Figure 11 describes the different types of MNs and approaches to drug transport through them into the skin.

### 9.6. Biodegradable Polymer Microneedle Arrays

Polymeric MNs are made from degradable polymers with controlled degradation rates, or from non-degradable polymers such as polydimethylsiloxane, polyacrylic acid, and polyvinyl methyl vinyl ether. In order to provide various mechanical features and functions, degradable MNs with varying swelling rates, degradation rates, and biological reactions can be prepared from natural polymers (e.g., hyaluronic acid, silk fibroin, gelatin) and synthetic polymers (e.g., polyvinylpyrrolidone, polylactic acid) [10,11,12,13]. Following the release of drug, the biodegradable polymers in the MN patches are absorbed into the skin, hindering the MNs from residing in the human body [8]. Degradable MNs are mainly used to regulate the release of different medications and bioactive substances. Degradable MNs derived from appropriate polymers can result in the sustained release of pharmaceuticals for months, as opposed to the fast release of the dissolving MNs [9].

Biodegradable polymer MN arrays are a modern class of MNs that are intended for TDD. These needles are composed of thermoplastic polylactic acid, which is a substance that can be recycled and is environmentally friendly, since it degrades naturally. They are manufactured through FDM, which increases their cost. Normal degradation can affect the resolution in the FDM method, which leads to problems in the tips of the MNs. This was solved by Micheal et al. (2018), who created a chemical etching protocol that follows the FDM method. This permits the manufacture of MNs with tips as small as 1 μm. The resulting needles were tested on pig skin, where the MNs easily pierced the skin. The distinctive features of stability and degradability enabled these MNs to be filled with small treatments and then discharged into the skin gradually [80]. The same results were obtained by Camović, Mirela, et al., (2018), who have confirmed that when 3D printing of MNs is followed by a post-fabrication etching step, this contributes to MNs with superior size and shape [181].

## 10. Factors Affecting the Mechanical Features of 3D-Printed Microneedles

Any 3D-printed MNs should comply with certain criteria to succeed as delivery systems—for instance, the ability to be inserted into the deep layers of the skin, with mechanical durability that prevents breakage, suitable dimensions and design, and utilizing a proper biocompatible molecule [182,183].

### 10.1. Substance Selection

The substances exploited in MNs must have the ability to withstand mechanical stresses and compression, to allow insertion deep into the skin. Moreover, stability, safety, biocompatibility, medication filling, stretchability, and flexibility are required in the MNs [153,154,184]. It is challenging to find materials and substances that have these properties and are also non-toxic. For instance, resins used in 3D printing include methacrylate-based resins, which are used excessively in the SLA fabrication technique [185,186,187]. In addition to acrylate-based resins [87,88,188], biocompatible polypropylene fumarate [189] and polyethylene glycol diacrylate [86] are commonly used in the DLP method, along with many others. A study conducted by Mansor et al. (2019) inspected numerous polymers that were considered as potential candidates to be used in the SLA method. They compared polylactic acid, polyvinyl alcohol, acrylonitrile butadiene styrene, and polyester resin in terms of the mechanical features required by MNs and their manufacturing process. They concluded that polyvinyl alcohol had the optimal mechanical strength to tolerate the energy added in the manufacturing procedure [190].

### 10.2. Precision of 3D Printing

Obtaining high-quality MNs is associated with analyzing and identifying the important factors in the procedure. Optimization of these factors is vital for refining the configuration and dimensions of the product, leading to improved accuracy [191]. To achieve the requested design of the MNs, with piercing tips, the printer needs to have high resolution. The mesh density, which is controlled by the printer’s software, has a direct effect on the accuracy of the printing machine. In addition to the results obtained by the printing machine, which manages the movement of each part in the printer—such as the nozzle, belts, thread spindles, and other components [192,193]—the key factors among the process parameters are as follows:

1. The sheet width, which has a chief impact on accuracy. This impact is particularly important in the construction of bent surfaces, owing to the distinct “staircase” phenomenon. Hence, by increasing the sheet width, the accuracy of the produced material is reduced, and the material is rough [85].
2. The dimensions of the needle should be carefully identified. If the MN is too lengthy or brittle, it will break during insertion into the skin [159,194]. For diagnostic purposes, the length of the needle should be no less than 900 µm in order for it to be able to absorb the interstitial fluid. If it was shorter, the skin would wrap around the needle during administration [159,195,196].

### 10.3. Microneedle Design

The dimensions and design of the MN have a direct effect on the MN’s performance, irrespective of the fabrication method. The dimensions should be ideal to achieve optimal insertion and skin penetration, in addition to the required rate of drug delivery. The main geometrical dimensions are the MN length, the base and tip width, and the core and shape of the MN [195,197]. MNs usually range between 150 and 2000 μm in length; these dimensions are necessary to enable the MN to perform its required task. The internal length of the bending part of the MN or the radius is fundamental to the MN’s design. It usually ranges between 50 and 250 µm for the core, while the tip dimensions are only about 1–25 μm. These measurements are vital for appropriate insertion and removal of the needle without breakage [121]. Moreover, these dimensions guarantee that the MN is inserted away from the nociceptors that are situated in the dermis [26,121].

Another important parameter is the sharpness of the tip. A sharp tip allows for good infiltration of the skin, with the lowest possible force of insertion [159,194]. Davis et al. (2008) compared different tip dimensions and angles of insertion in vivo, measuring the force required for inserting the MNs. The team concluded that higher wall width and higher tip angle and width increased the strength needed for the MNs’ insertion [198]. They also compared multiple angles with different bevel angles and identical wall thickness. The results showed that the lower the MN angle used, the less penetration strength was required [199]. The density of the MN can also play a significant role in skin penetration [186]. The “Bed of nails effect” is a phenomenon that takes place when the density is high, and this also negatively affects the insertion [200].

The shape of the MN—which is either conical, pyramidal, or cylindrical—has an effect on the MN’s performance. As was discovered by Pere et al. (2018), conical MNs need less strength to pierce experimental pig skin models than the force required with pyramidal shapes [11]. However, Economidou et al. (2021) concluded that the sharpness of the MN is more important than the shape, with all shapes being suitable for administration [194]. Yeung et al. (2010) also compared three needle shapes: pyramidal, conical, and fine-tip syringe-shaped. The three shapes were fabricated using the same technique (SLA), and the evaluation was through insertion into parafilm layers. The results showed that the best piercing ability was obtained by syringe-shaped MNs, which left marks to the second parafilm layer, while the pyramidal and conical shapes left marks only on the first layer of the parafilm [162].

The shapes are affected by the type of manufacturing method used. The results of two studies conducted by Sirbubalo et al. (2021) and Camović et al. (2019) showed difficulties in achieving sharp tips with seven shapes through the FDM 3D printing technique, due to the reduced resolution of the printing nozzle, even after alkaline treatment [160,181]. However, in a study conducted by Tang et al. (2020), the same method or printing technique resulted in sharp conical MNs [81].

## 11. Assessment of MNs

### 11.1. Physical Characterization

All of the important parameters—such as the geometry, dimensions, surface morphology, and distribution—of MNs on the array must be assessed. The available techniques for assessment are visual inspection, scanning electron microscopy, and stereomicroscopy [27]. Drop shape analysis and contact angle determination are used to determine the characteristics of the superficial area of MN patches [90]. The drop shape analysis method uses measurements of the connection angle fluid drop, and images are taken and analyzed through equations [201]. MN patches can be identified through fluorescent labelling or any other coloring method that can be detected through fluorescent microscopy, as well as by confocal laser scanning microscopy or visual assessment. Visualization is important for specifying the location of substances integrated in the MN patch, whether it is the backing layer, the shaft, or the tip [202]. Coated MNs are assessed through FTIR spectroscopy [90].

### 11.2. Mechanical Characterization

MNs are subjected to numerous stress conditions during application because of the lack of uniform skin surfaces, along with sudden movements and the mechanical tension applied on the MN once it is taken off the skin [203]. It is vital to perform mechanical characterization of the critical features of MNs, since this provides certainty for the safety of the product. MNs have a tendency to bend, break, and buckle when injected transdermally, because of their lack of flexibility. Mechanical characterization can be performed using a wide collection of tests that simulate the conditions of skin penetration in vivo [121]. The following are some of these tests.

#### Failure Force Tests

These tests are essential in determining if the MN has adequate mechanical strength to endure distortions and changes throughout application into the skin.

A. Axial Fracture Force Tests

**Figure 12:**
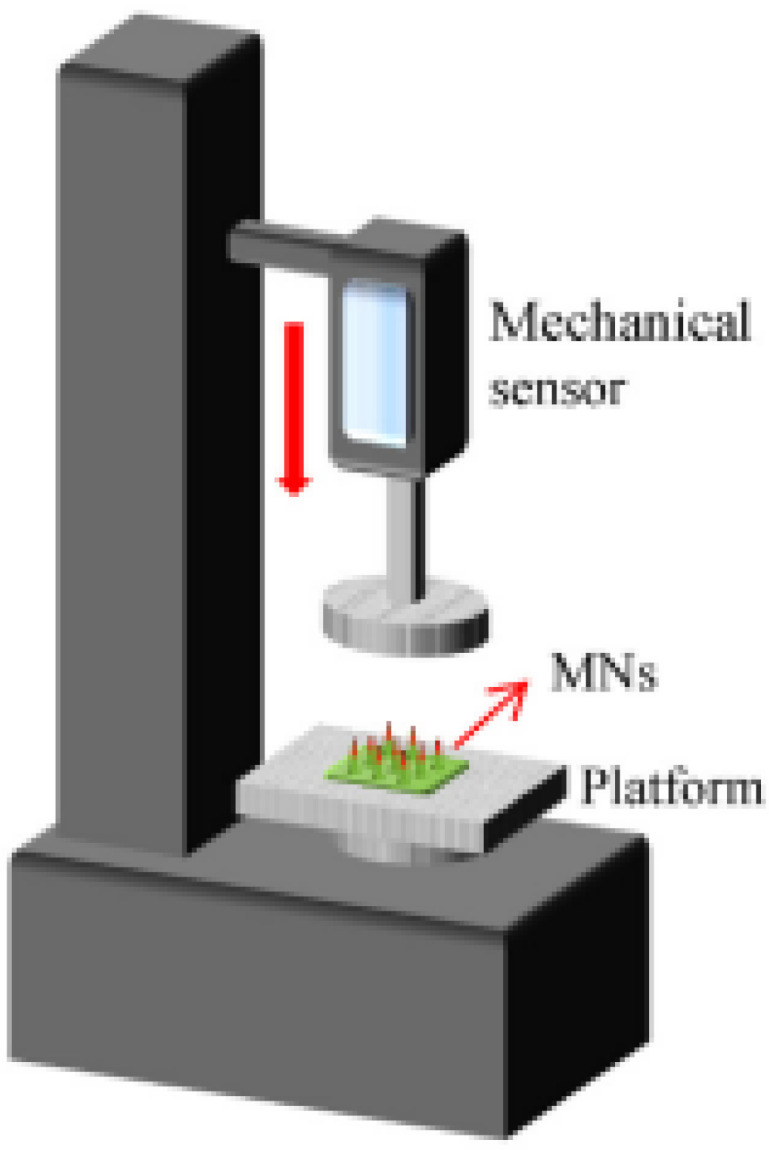
Schematic representation of a texture analyzer [151]. Reprinted/adapted with permission from Ref. [151]. 2018, Qi Lei Wang, Jia Wei Ren, Bo Zhi Chen, Xuan Jin, CanYang Zhang, Xin Dong Guo.

This kind of test estimates the malfunctioning of MNs due to both transverse and axial loading. This test is performed by placing pressure on the MN array parallel to a firm metal surface. The test continues to quantify the force and dislocation while producing pressure against the strain curves (Figure 12). When MNs fail, the force reduces abruptly, and the highest force exerted just before the drop represents the force of the MNs’ failure. To define the failure style, the MNs are viewed using a microscope, and the images are compared to ones taken prior to the failure [204]. Axial fracture force tests utilizing single MNs should be elucidated carefully, since their outcomes might not correlate with images obtained from an MN array [205]. It should be considered that the applied force on the MNs throughout the compression studies is variable and inaccurate in comparison with penetration into the skin.

Compression studies include pressing of MNs against a solid metal surface, wherein the whole applied force is focused on the connection surface of the MN tip. The forces utilized in skin penetration by MNs are distributed over a larger MN area, particularly after primary inclusion, as the elastic skin wraps around the MNs [206].

B. Transverse Fracture Force

These kinds of tests are used to evaluate MNs’ usage. This test is performed on single MNs or on a row MNs, wherein the applied force can be divided by the number of MNs in the row in order to estimate the transverse fracture force per MN. The main drawback of this test is the need for manual orientation of the metal probe with a definite size, which might lead to inaccurate measurements [33].

C. Baseplate Strength and Flexibility Tests

Breakage of the baseplate while the MN is inserted by the patient is to be avoided; thus, the strength/breaking force needs to be evaluated. Flexibility is an important parameter that plays an essential part in the MNs piercing into the skin, and it should be adequate for them to penetrate into different skin types without breakage. The test is performed by placing the baseplates between two blocks made from aluminum, and force is then applied to a metal probe. Thus, the force needed to break the baseplate can be calculated by noting the maximum peak value on the force–distance curve. The baseplate’s flexibility can be measured from its bending upon fracture [33].

D. Insertion Force Tests

Defining the MNs’ insertion force enables the estimation of the suitable length of the MNs. Calculating the insertion force needed for the MNs to penetrate the skin is vital for assessing fracture forces.

The force required for fracturing the needle should be much greater than the force needed for the insertion of the MNs into the skin. The insertion process of MNs is performed either through applicators or manually. However, applicators provide better control of the insertion surroundings and lessen the available inconsistency in comparison with insertion without applicators [121]. Usually, the insertion force must be around 0.098 N/needle to be able to penetrate the skin [207]; however, researchers have shown that only 0.03 N/needle is adequate [208].

Histological cryosectioning is a method in which the skin that is treated with MNs is taken away and frozen with liquefied nitrogen. In order to facilitate the identification of the channels formed by the MNs, a dye is used—generally hematoxylin and eosin. The depth of the cutaway tissue ranges between 6 and 12 µm [209]. The drawback of this method is that it is an invasive method, in addition to the overestimation that can be registered due to the alterations that can occur in the samples with regard to hydration and elastic tension [210].

Confocal laser scanning microscopy is a technique that enables the calculation of the measurements of minute holes produced by piercing MNs. The skin sample is exposed to treatment with a florescent dye, which enables the calculation of the thickness of the channels made by the MNs. A disadvantage of this method is the inability to quantify MNs longer than 250 µm, since it only detects ranges between 200 and 250 µm [192,193]. In addition, it is being dependent on the degree of clearness of the MNs, since opaque MNs (e.g., silicon, colored polymers, or metals) need to be treated first, prior to obtaining the photos, which might result in contraction of the holes, leading to inferior results. However, this is considered to be a non-invasive method [203].

Optical coherence tomography can detect the needles’ dimensions accurately, without being invasive. A semi-empirical model was created, derived from the findings of seven distinct water injection tests into pig skin tissue. The model used assumed a spherical fluid flow and tissue deformation and forecasted the flow rate over time using optimal experimental data and model parameters. It was based on the short coherence length of wide-band light sources that execute images of very small dimensions (micrometer-scale). This approach achieved great success due its many advantages, including the follows: (1) High-quality images due to a small range that lies within 1–10 μm axial resolution, and sometimes smaller—to a level of sub-micrometer (0.5 μm) resolution. (2) Fast imaging—for instance, a temporal image can be taken within a speed of milliseconds. (3) Label-free imaging, which means that the optical coherence tomography imaging can provide high-resolution images without the need for a contrast dye. (4) Cost-effectiveness. (5) Sophisticated functions can be added to the technique, such as the imaging of blood flow by Doppler optical coherence tomography [211,212]. This method has been widely used in the imaging of soft tissue; thus, it has been employed in ophthalmology, cardiology, gastroenterology, urology, dermatology, dentistry, and neuroscience [212].

An archetypal optical coherence tomography system comprises a low-coherence wide-band light source. The radiated light is joined with an interferometer. The light is allocated into two arms: a reference arm and a sample arm. The reference arm conducts the light in the direction of a reference mirror. The sample arm directs the light into the required tissue. The sample arm encompasses an objective lens that concentrates the light onto the sample tissue, such as the brain or the retina. The light that is backscattered from the tissue assemblies is rejoined with the reference light that is reflected from an extremely reflective (>95%) moving reference mirror, generating an interference pattern that can be detected using a light detector. Building two-dimensional (2D) or 3D cross-sectional items, the stream of light is scanned through the sample surface. Sophisticated systems might contain a charge-coupled device camera and diffraction grating [212], in addition to having the ability to unify the results of penetrability—which are different between human and animal studies [213]—and being able to perform at various tension levels on different skin models. However, the MNs also need to be translucent [214].

X-ray transmission computational tomography is a modern process that is also non-invasive and uses a number of scans taken at multiple angles, which permits 3D imaging of MNs’ geometry. A disadvantage of this procedure is that it only works on materials that are suitable for X-ray imaging, such as metals. Skin layers that are pierced cannot be easily differentiated using this method [27,215].

### 11.3. Skin Irritation and Recovery Studies

Slight and momentary arrhythmia occasionally occurs when using MNs, which depends on the type of therapy, used along with their size. This can be measured by a technique called the Draize method. A microscope is used to detect the changes that occur on the skin after treatment, through comparing erythema and edema before and after treatment with MNs [216].

#### Permeation Studies

A. In Vitro Studies of Permeation

In vivo tests are implemented on either human or animal skin models to determine the quantity of therapeutic agent received by the cells through diffusion. The therapeutic molecule is introduced into the donor compartment and, through membrane diffusion, is transported to the receptor compartment [217].

Transdermal transport of a certain molecule through MNs can be confirmed through implementing sampling of the receptor compartment and analyzing the sample via high-performance liquid chromatography (HPLC). Moreover, extraction analysis methods can be applied to detect intradermal distribution of a specific molecule from the skin model after penetration [27]. The available models of diffusion cells are the following: (1) The static model (Franz type), which is further classified according to the skin’s alignment inside the diffusion compartment into lateral and vertical side-by-side and upright cells. Lateral diffusion cells are no longer used in studies because of the overestimation of permeation that takes place because of extreme and lengthy moisturization of the skin located in the donor compartment, in addition to the requirement of soaking the SC tissue in the solutions of the donor and recipient compartments, which injures the skin. Skin loss should be taken into consideration when skin permeation studies employ the Franz cell type [218,219]. (2) The flow model (Bronaugh type), where constant flow is sustained by a pump that ensures the solution’s flow through the receptor chamber. The flow simulates the circulation in the skin layers [220]. The Franz cell flow model method is complex and expensive [221,222].

B. In Vivo Studies of Permeation

In vivo studies are performed to evaluate the TDD with regard to the absorption, disposition, and permeation of molecules administered through MNs. Choosing a suitable in vivo model depends on some important features, such as the depth and flexibility of the skin. Pig skin is the model of choice in in vivo TDD studies, despite the variation in structural, histological, and morphological properties between humans and pigs. Rat skin is sufficiently suitable for in vivo TDD studies, especially given that rats are less expensive and easier to handle than pigs, although their skin can provide higher permeability than human skin [223,224].

## 12. Applications of 3D-Printed Microneedles

MNs have been used in TDD for immunobiological administration of vaccines and antibodies, disease treatment and diagnosis, and cosmetic use. The following are some of the applications of MNs.

### 12.1. Disease Treatment

This is one of the most common applications of MNs, especially for biological agents, peptides, proteins, hormones, and natural agents that cannot be given orally due to first-pass metabolism. Thus, hypodermic administration represents a solution.

#### 12.1.1. Cancer

Cancer chemotherapy has a lot of severe side effects, and one of them is the severe pain associated with the injections. Thus, cancer treatments always provide a wide area for research. MNs can offer a solution to this problem, since they are considered to be negligibly invasive, painless, applicable, and user-friendly [225]. For instance, a dissolving MN array containing the chemotherapeutic agent doxorubicin, hyaluronic acid, and gold nanocages was synthesized to treat superficial skin tumors. The loaded gold nanocages served as effective photothermal agents for transdermal therapy for superficial skin tumors, as well as serving as reinforcements to increase the mechanical strength of the MNs. The resulting MNs had excellent skin penetration, skin dissolution, and cargo-release capabilities. Tumors were effectively eliminated through a synergistic combination of doxorubicin and the gold nanocages produced by near-infrared laser irradiation. Furthermore, the strong antitumor effects of the doxorubicin-/gold-nanocage-loaded MNs were confirmed after four clear-cut administrations to mice bearing superficial skin tumors. As a result, the drug-/gold-nanocage-loaded dissolving MN system offered a promising framework for the combined treatment of superficial skin tumors that was efficient, secure, and minimally invasive [225].

Immunology in cancer is now extensively studied, and immune-checkpoint inhibitors are considered to be a potential cancer treatment [226,227]. Moreover, a supporting array of dissolvable polyvinyl alcohol/polyvinylpyrrolidone that delivered photosensitive nanoparticles (lanthanum hexaboride) and doxorubicin evenly heated the target tissue to create an extensive thermal ablation area when subjected to near-infrared light. Then, doxorubicin was released over a wide area, eliminating tumors. It was proven that after only one MN usage and a total of three sessions of treatment with lasers, 4T1 tumors were eliminated completely within a week. Neither recurrence of the tumors nor any significant loss in body weight in the mice was seen [228].

A hollow MN system was achieved with a large synthetic peptide that works as a cancer vaccine, since it enhances T-cell reactions against cancer. The difference between TTD and intradermal injections is that the amount needed in TDD is less than what is required in intradermal injections, improving the efficacy of the vaccines’ immunogenicity and the effectiveness of tumor vaccine formulations [229]. Hindrance of tumor growth and activity was seen after Tang et al. (2016) used an MN array containing small interference RNA that inhibited certain genes related to cancer growth [230]. All of these studies were conducted using MNs fabricated by conventional methods.

On the other hand, many studies have used 3D printing for manufacturing MNs used for the delivery of cancer drugs, such as the study conducted by Uddin et al. (2020), who were able to design a polymeric MN array using SLA and inkjet dispensing to coat the MNs with the oncology product cisplatin. The MNs allowed the delivery of 80–90% of cisplatin over the course of 60 min. In vivo studies confirmed a good response to cisplatin using the MN array, through observing tumor regression. These MNs could be loaded with different chemotherapeutic agents. This new treatment approach gave hope for cancer patients [231]. Bhatnagar et al. (2017) prepared zein MNs made from the natural material corn protein through 3D printing techniques (STL). A micromolding procedure was used to prepare a cone-shaped cast. The MNs were tested for antigen transport with albumin. The histological studies proved that the transport of antigen/albumen complexes through zein MNs and the bacterial contamination during the MNs’ penetration were much lower than with conventional syringes. An additional feature of antigen/albumen zein MNs was their stability at both ambient and refrigerated temperatures. The most important characteristic of these MNs was their success in immunization, where antibody titers (total IgG, IgG1, and IgG2a) were higher in comparison with the conventional syringes. The zein MNs showed great potential for future vaccination [232]. Again, Bhatnagar et al. (2018) used 3D printing to create zein MNs through micromolding technology, which uses a cast and prints a main mold as a master. The polymer employed in the cast was acrylobutyl nitrile styrene. Plasticizers were added, such as glycerol and PEG 400. The breast cancer therapeutic agents tamoxifen and gemcitabine were loaded into the MNs as a coating. The results showed that tamoxifen must be added in greater quantities than gemcitabine, since gemcitabine had higher permeation than tamoxifen. The pharmacokinetic studies proved that zein MNs facilitated better permeation [233]. Uddin et al. (2020) prepared 3D-printed polymeric MN arrays for the treatment of cancer that enhanced the delivery of cisplatin to A-431 epidermoid skin tumors. The SLA method of selective photopolymerization of successive layers of a biocompatible photopolymer resin was used to create the MNs, which were then coated with cisplatin formulations by inkjet dispensing on the needles’ surface. To enhance the mechanical and optical coherence of the MNs, the printability via SLA was improved. Tomographic analysis revealed that 3D-printed MNs had an excellent piercing ability to a depth of 80%. In vivo testing on mice showed adequate cisplatin permeabilization, with high anticancer activity and tumor regression. Franz cell diffusion studies showed rapid cisplatin release rates of 80–90% within 1 h [231].

**Figure 13:**
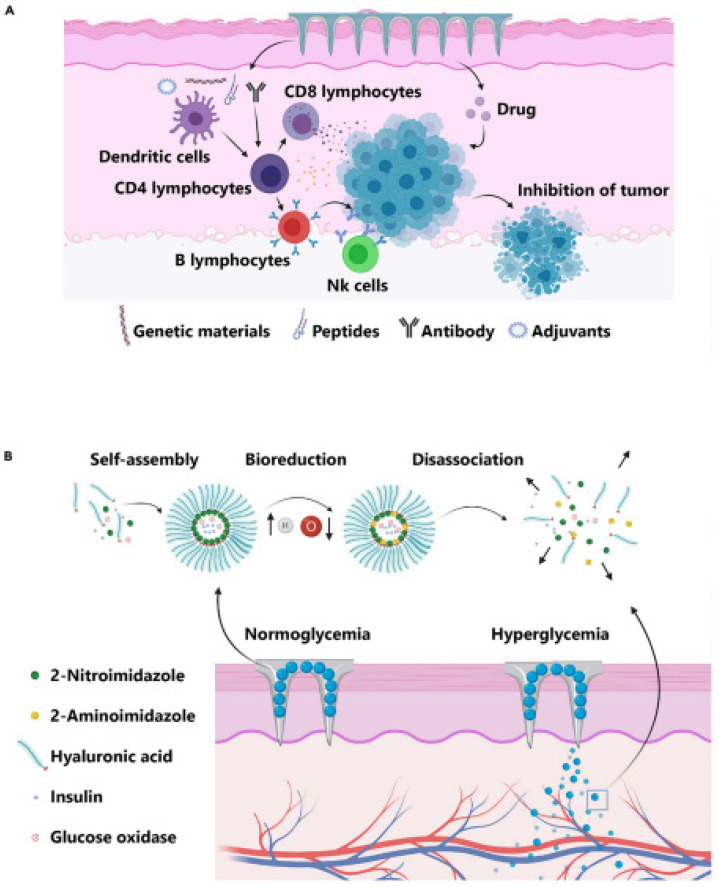
Multiple uses of MN patches in oncology: (**A**) The incorporation of antibodies and peptides. (**B**) Intelligent needle assembly [112].

Figure 13 shows various uses of MN patches in cancer treatment.

#### 12.1.2. Diabetes

Diabetic patients’ compliance mainly relies on providing a less painful needle, especially considered that the patient is sometimes required to inject themselves multiple times per day. In response to this problem, smart insulin MN array patches that manage blood glucose levels in type 1 diabetic patients were developed. They rapidly responded to glucose levels, and they were painless [234]. In a clinical study, using a 3D printing technique, polymeric MN batches were manufactured through SLA; the aim of the MNs was to deliver insulin through TDD. After the MNs were constructed with the aid of a resin, coating was performed with a mixture of insulin, trehalose, and xylitol (the latter two were used as preservatives, carriers, stabilizing agents, and release facilitators for insulin). It was confirmed by this study that the insulin release rate from the polymeric MNs was complete in 30 min; thus, 3D printing of MNs through SLA proved to provide superior features of the resulting MNs [11]. A similar study conducted by Economidou et al. (2019) confirmed the results provided previously by Pere, Cristiane Patricia Pissinato, et al. (2018), who had also designed MDs through SLA using a biocompatible resin for TDD of insulin. The formula consisted of insulin, a disaccharide, a biocompatible resin, and an alcohol. The layers were added successively. The skin permeation was greater than with needles manufactured by conventional methods, in addition to higher speed of action of insulin in cases of high blood sugar, and accompanied by a steady state of blood glucose levels [186].

Wu et al. (2020) conducted a study employing the extrusion-based 3D printing technique to produce a microneedle patch. These MNs were intended for the painless supply of insulin to patients with diabetes mellitus, in response to high glucose levels. To fabricate the MNs, comprehensive examination of suitable bionics was performed, using the additive substance alginate hydroxyapatite. The tips of the MNs in the patch array were formed in a cylindrical shape, whereas the base was a conical shape. These MNs were tested on mice and showed good mechanical strength, since they were able to pierce the mice’s skin without being damaged. This also proved that these MNs had good potential to be used in TDD, as they maintained the glucose serum concentrations in mouse models of type 1 diabetes within normal glucose levels for 40 h, when given one time only. Additionally, they improved the symptoms of diabetes [195].

### 12.2. Immunobiological Administration

Vaccines are usually administered through intramuscular injection or intradermal injection. However, some people suffer from a phobia of needles, and MNs are a solution for this drawback. The delivery of vaccines through MNs has been widely studied and is finally in practice [7,235]. Many vaccines have been made using conventional methods, such as in the following studies: A newly developed vaccine for hepatitis B that employs an MN array is now available in the market [236]. Additionally, an MN array that contains cholera toxins is also available, and with a better profile than the intramuscular injection [237]. The use of dissolving MNs reduced the penetration time of monoclonal antibodies using maltose from 24 h to 1 min [237]. Moreover, dissolving MNs enabled the delivery of potent hormones and other organic molecules in very small amounts [97]. An influenza virus vaccine was improved by Sullivan et al. (2010), who increased the immunity resulting from the vaccine by adding a biocompatible polymer with the MNs [238]. Furthermore, vaccine stability was recently enhanced by Raphael et al. (2016) by making adjustments with mannitol, sucrose, trehalose, and sorbitol [239]. A ceramic and nonporous MN array proved to provide enhanced delivery of diphtheria toxoid and tetanus toxoid compared to conventional needles [240].

MNs are utilized in vaccination, which is extremely important in preventing pandemics; thus, painless injections are required to encourage people to take the vaccines. In a very recent study, 3D printing using the CLIP technique was exploited to fabricate a faceted MN, which was characterized by having an enhanced surface area in comparison with pyramidal structures. In mouse models, a comparison between the conventional injections and MNs was made to evaluate the delivery of the vaccine and the resulting immune effect. It was concluded that MNs not only had a higher load retention time, but also led to higher humeral immune activation and increased the total number of immunoglobulins and IgG. Moreover, the MNs enhanced the numbers of T cells, cytotoxic CD8+ T cells, CD4+ cells, and cytokines [241].

### 12.3. Cosmetic Field

Recently, MNs have been excessively employed in the cosmetics field and achieved great success. The main aim of cosmeceuticals is to deliver different molecules into the skin with the least amount of injury. These molecules could be employed either to enhance wound healing or to increase the permeation of pharmaceuticals [242]. MNs have many advantages over other methods, such as inducing less erythema and post-inflammatory hyperpigmentation after the removal of marks with a laser [243]. MNs also have the benefit of providing safe and effective delivery of cosmeceuticals through microchannels, without the probability of hurting a facial nerve. MN patches containing retinyl retinoate and ascorbic acid had a positive effect on wrinkles and were devoid of undesirable effects such as allergy [244]. In cosmeceuticals, there has been an upsurge in the MN industry, and their uses can be classified into two main categories: The first is patches, which suffer from the disadvantage of needing to be inserted manually into the skin, which leads to a low penetration effect. This can be solved by using an intense-speed applicator, which overcomes the rigorousness of the skin and allows for better skin permeation [245]. The second category is MN rollers, such as dermaroller^®^. These are superior to MN patches in terms of their efficiency in penetrating the skin. Newer rollers are manufactured by 3D printing techniques and also emit light at a certain wavelength; one of these devices is DermaFrac™ [61].

Lim et al. (2021) developed a delivery system for a small peptide with an anti-wrinkle effect by employing the DLP 3D printing technology. Acetyl-hexapeptide 3, which is the peptide used in the novel system, is known for its safety and efficacy as an anti-aging molecule. Optimization of the resins—mainly vinyl pyrrolidone and polyethylene glycol diacrylate—was performed by examining various concentrations of the polymer and evaluating their effect on important factors such as the swelling rate of the polymer and its durability. The developed MN patch exhibited sufficient capacity to permeate the human skin, while maintaining its shape integral after being stressed. Additionally, the polymer was safe to the dermal fibroblasts, making MNs a potential future anti-aging product [246].

### 12.4. Examples on Various MN Arrays for TDD Systems Based on 3D Printing

Since the release of Spritam^®^—the first 3D-printed drug to receive FDA approval—in 2015, this technology has advanced through in-depth research. Some examples of different biomaterials and 3D printing techniques that have been used to create MN patches for TDD applications are presented in Table 2. Further clinical studies are needed to evaluate the efficacy and safety of the 3D-printed MNs, since no studies have been conducted yet. To date, no patents for 3D-printed MNs have been registered, due to the lack of clinical trials.

## 13. Conclusions and Future Perspectives

Regardless of knowing that existing MN manufacturing processes could produce an acceptable resolution, the constraints of traditional techniques already exist. These include labor-intensiveness, the need for manual steps, the requirement for high proficiency in micro-manufacturing for proper implementation, and cost-effectiveness. The ability to directly design, modify, and fabricate proposed MNs with desired size features using 3D printing shortens the design and prototyping process by doing away with the need for outside manufacturing firms. However, there are significant drawbacks to 3D printing, including slow printing, resolution restrictions, a small material selection, and biocompatibility [34,256].

The extraordinary comfort and accuracy of 3D-printed MNs and the many types of MNs that 3D printing allows to be fabricated—with reasonably low price, high accuracy, and specific aims or patient design—have made 3D printing revolutionary. The various manufacturing methods of 3D printing and the ability to use multiple inks and raw materials, including biocompatible substances, has allowed an upsurge in the utilization of 3D printing in different fields, such as the pharmaceutical, cosmeceutical, and medical fields. This technology has played a huge role in optimizing the TDD dosage forms, through permitting the manufacturing of numerous types of MNs for the administration of multiple molecules, such as insulin and vaccines.

Despite the terms “3D printing” and “rapid-prototyping” having been used interchangeably, the actual 3D printing process is slower than traditional industrial approaches such as injection molding [34]. Furthermore, despite the considerable attention that 3D printing has received, obtaining higher resolutions remains difficult. SLA, TPP, and direct laser writing may generate MNs with acceptable resolutions (5 μm), but they only support a small number of biocompatible materials, and their mechanical properties are still not that good compared to conventional methods [34,47,48,49].

There is continued work to find solutions for the current drawbacks in the 3D printing manufacturing techniques, as well as continuous developments in the available characterization and analysis procedures.

Future research may therefore focus on developing 3D printing technologies that are faster, without sacrificing resolution. The ultimate resolution of printed MNs can be achieved by improving the laser beam features in laser-based methods and nuzzle features in extrusion-based methods. Moreover, many studies could be conducted to develop other kinds of MNs used in different applications. Furthermore, clinical trials are needed for determining the safety and efficiency of 3D-printed MNs, because to date there have been no clinical trials conducted on these kinds of MNs, since all of the studies have been conducted on animals and mimicking tissues. There have also been no patents filed regarding 3D-printed MNs. Additionally, since many drugs exhibit restricted solubility in polymer compounds (especially in polymeric MNs), the properties of the drugs might result in certain limitations. Moreover, when exposed to high temperatures or UV rays throughout the printing process, drugs may be absorbed. Thus, new materials and polymers must be used to fabricate different types of MNs in order to enhance drug-loading capabilities. As a result, to improve the quality of TDD systems and encourage their commercialization, new 3D printing systems must be developed in addition to the existing ones. Another issue at this stage is the commercialization of findings. Therefore, solid scientific foundations and effective commercialization plans should be combined to speed patients’ access to this promising technology.

Microneedle printing using 4D technology is another potential future pathway. MNs with greater mechanical strength, tissue adherence, performance, and controlled drug delivery have been created using the 4D printing technique, used for biosensing, wound healing, and other purposes. Thus, in future, hypodermic needles might be replaced with 3D- or 4D-printed MNs [257,258,259].

## Figures and Tables

**Table 1:** Benefits and drawbacks of various 3D printing methods.

Printing Techniques (Resolution)	Benefits	Drawback	Materials Used	Reference
FDM (0.1–0.3 mm)	Simple, not expensive for thermoplastic materials; high speed; create complex, customized, innovative dosage forms	Expensive for both metal and glass; temperature fluctuations; weak mechanical characteristics; limited material range; the finishing is layer-by-layer; risk of API degradation due to high temperature during the extrusion; lack of both biodegradable and biocompatible printable polymers	Thermoplastic polymers; metal and glass forming a continuous filament.	[108,109,110]
SLA (0.025–0.125 mm)	Simple; fine spatial resolution; low costs; high quality for customized and complex drug delivery systems	Limited mechanical characteristics for the produced product; low availability of the polymers; UV light is needed to start the polymerization (degradation for API); toxicity; post-curing and rinsing is required	Liquid photopolymer	[108,109]
DLP (0.012–0.2 mm)	High resolution, speed, and low cost	Needs support; possible toxicity; limited by pixel size; reduced ability of the machine upon continuous usage	Epoxides; acrylates	[108,109]
LCD (0.05–0.1 mm)	High resolution: short curing time and low cost; using resins with small volume	Precision is low	UV-curable resins	[89]
CLIP (0.05–0.1 mm)	Fastest 3D printing technique; high precision; constant manufacturing	Most costly; possible toxicity; heat dissipation of dead zone; unsuitable for oxygen-insensitive substances	Acrylates; UV-curable resins	[108,109,110,111]
2PP (100 nm–5 µm)	Low material costs; high resolution	Low yield of production; low build speed; limited materials can be used	UV-curable resins; acrylates; ceramics	[67,93,98,112,113]
SLS (20–150 µm)	Support material is not needed; high resolution and precision (30 µm); fast process with high precision; no post-curing process required	Restricted mechanical properties; expensive; slow printing process; high temperature is produced during printing; rough surface	Thermoplastics; polymers; metals; ceramics	[49,114,115]
DMLS (20–100 µm)	High resolution; good mechanical characteristics; good accuracy	The need for support structures; a protective atmosphere	Alloys; metals as a compact powder (fine)	[103,116,117]
SLM (20–100 µm)	Good mechanical characteristics, support material is not needed	High cost; poor dimensional accuracy; poor quality	Alloys; metals	[49,114,116]

**Table 2:** Examples of different 3D printing methods used in the production of MN arrays for TDD systems.

3D Printing Method	Type of Microneedle Produced	Drug Model/Medical Agent	Material Used for Fabrication	Main Observations	References
Inkjet printing	Coated metal MNs	Insulin	Gelatin, polyvinyl caprolactame-polyvinyl acetate-polyethylene glycol, poly(2-ethyl-2-oxazoline), and trehalose	Rapid release rates for insulin were observed within the first 20 min; poly(2-ethyl-2-oxazoline) and gelatin were rapidly released from Franz diffusion cells from MNs implanted into porcine skin	[247]
	Coated Gantrez 169 BF MNs	Amphotericin B	-------	In a radial diffusion assay, controlled release of amphotericin-B was found to be effective against *Candida parapsilosis*	[248]
	Coated biodegradable polyglycolic acid MNs	Voriconazole	Polyglycolic acid MNs	MNs modified with voriconazole exhibited antifungal activity against *Candida albicans*, but not against *Escherichia coli*, *Pseudomonas aeruginosa*, or *Staphylococcus aureus*	[249]
	Coated metal MNs	5-Fluororacil, curcumin, and cisplatin	Hydrophilic graft copolymer Soluplus^®^	A fast release rate ranging from 3 h for 5-fluororacil to 1 h for curcumin and cisplatin throughout the highly precise coatings at different drug–polymer ratios in the produced MNs	[250]
DLP	Dissolving MNs	Gold/silver nanoclusters	Polyvinyl alcohol/sucrose MNs	Gelatin with gold/silver nanocluster labels functioned as a fluorescent probe; DLP was used to develop an effective polyvinyl-alcohol-based MN patch; the skin patch could be easily removed to allow for additional nanocluster release	[251]
SLA	Biodegradable MNs	Dacarbazine (anticancer drug)	Dacarbazine-loaded poly(propylene fumarate) MN arrays	The controlled release rate for the drug extended to 5 weeks	[189]
FDM	Biodegradable MNs	Fluorescein	Polylactic acid	Fast printing of polymeric MN via customized needles; this polylactic acid was used to load small-molecule medications	[80]
Two-photon polymerization (2PP) 3D printing	Hollow MNs	-------	Silicone	MNs with sufficient stability within skin tissue	[252]
Magnetic-field-assisted 3D printing	Biodegradable MNs	Fluorescein	Iron oxide nanoparticles encapsulated by photocurable E-glass resin	Fluorescein was reported to be released continuously from nanocomposite MNs, which could be inserted into the skin painlessly	[253]
Continuous liquid interface production (CLIP)	Biodegradable MNs	Fluorescent	Trimethylolpropane triacrylate, polyacrylic acid, and photopolymerizable derivatives of polyethylene glycol and polycaprolactone	The development of square pyramidal MNs made of different kinds of polymers; the MN patch was able to release the fluorescent drug surrogate and successfully penetrate murine skin	[254]
PolyJet 3D printer	Biodegradable MNs	Ovalbumin (model antigen)	Corn protein and zein	Zein MNs with a cast cone shape were successfully created; when compared to the application of a hypodermic syringe, considerably lower bacterial penetration through the skin was seen	[232]
Magnetorheological drawing lithography	Dissolving MNs	Rhodamine B	Polyvinyl alcohol and sucrose	MN arrays in the shape of cones were developed effectively on a flexible PET substrate; the patch demonstrated good strength, along with excellent and easy skin penetration; due to the MN patch’s creation of microchannels, drugs may be dispersed through the skin	[255]

## Data Availability

Not applicable.

## References

[1] Yan L., Alba M., Tabassum N., Voelcker N.H. (2019). Micro-and Nanosystems for Advanced Transdermal Delivery. Adv. Ther..

[2] Meng S., Zhang C., Shi W., Zhang X.W., Liu D.H., Wang P., Jin Y. (2016). Preparation of osthole-loaded nano-vesicles for skin delivery: Characterization, in vitro skin permeation and preliminary in vivo pharmacokinetic studies. Eur. J. Pharm. Sci..

[3] Iliescu F., Dumitrescu-Ionescu D., Petrescu M., Iliescu C. (2014). A review on transdermal drug delivery using microneedles: Current research and perspective. Ann. Acad. Rom. Sci. Ser. Sci. Technol. Inf..

[4] Jin X., Zhu D.D., Chen B.Z., Ashfaq M., Guo X.D. (2018). Insulin delivery systems combined with microneedle technology. Adv. Drug Deliv. Rev..

[5] Lee J.W., Park J.H., Prausnitz M.R. (2008). Dissolving microneedles for transdermal drug delivery. Biomaterials.

[6] Chang H., Zheng M., Yu X., Than A., Seeni R.Z., Kang R., Xu C. (2017). A swellable microneedle patch to rapidly extract skin interstitial fluid for timely metabolic analysis. Adv. Mater..

[7] Moffatt K., Wang Y., Singh T.R.R., Donnelly R.F. (2017). Microneedles for enhanced transdermal and intraocular drug delivery. Curr. Opin. Pharmacol..

[8] Hwa K.Y., Chang V.H., Cheng Y.Y., Wang Y.D., Jan P.S., Subramani B., Wang B.K. (2017). Analyzing polymeric matrix for fabrication of a biodegradable microneedle array to enhance transdermal delivery. Biomed. Microdevices.

[9] Hong X., Wei L., Wu F., Wu Z., Chen L., Liu Z., Yuan W. (2013). Dissolving and biodegradable microneedle technologies for transdermal sustained delivery of drug and vaccine. Drug Des. Dev. Ther..

[10] Dharadhar S., Majumdar A., Dhoble S., Patravale V. (2019). Microneedles for transdermal drug delivery: A systematic review. Drug Dev. Ind. Pharm..

[11] Pere C.P.P., Economidou S.N., Lall G., Ziraud C., Boateng J.S., Alexander B.D., Douroumis D. (2018). 3D printed microneedles for insulin skin delivery. Int. J. Pharm..

[12] Xie L., Zeng H., Sun J., Qian W. (2020). Engineering microneedles for therapy and diagnosis: A survey. Micromachines.

[13] Wang M., Hu L., Xu C. (2017). Recent advances in the design of polymeric microneedles for transdermal drug delivery and biosensing. Lab Chip.

[14] Chambers R. (1921). Microdissection studies, III. Some problems in the maturation and fertilization of the echinoderm egg. Biol. Bull..

[15] Gerstel M.S., Place V.A. (1976). Drug Delivery Device. U.S. Patent.

[16] He X., Sun J., Zhuang J., Xu H., Liu Y., Wu D. (2019). Microneedle system for transdermal drug and vaccine delivery: Devices, safety, and prospects. Dose-Response.

[17] Henry S., McAllister D.V., Allen M.G., Prausnitz M.R. (1998). Microfabricated microneedles: A novel approach to transdermal drug delivery. J. Pharm. Sci..

[18] Bevers T.B. (2001). Breast cancer chemoprevention: Current clinical practice and future direction. Biomed. Pharmacother..

[19] Mikszta J.A., Alarcon J.B., Brittingham J.M., Sutter D.E., Pettis R.J., Harvey N.G. (2002). Improved genetic immunization via micromechanical disruption of skin-barrier function and targeted epidermal delivery. Naturemedicine.

[20] McAllister D.V., Wang P.M., Davis S.P., Park J.H., Canatella P.J., Allen M.G., Prausnitz M.R. (2003). Microfabricated needles for transdermal delivery of macromolecules and nanoparticles: Fabrication methods and transport studies. Proc. Natl. Acad. Sci. USA.

[21] Miyano T., Tobinaga Y., Kanno T., Matsuzaki Y., Takeda H., Wakui M., Hanada K. (2005). Sugar micro needles as transdermic drug delivery system. Biomed. Microdevices.

[22] Bhatnagar S., Dave K., Venuganti V.V.K. (2017). Microneedles in the clinic. J. Control. Release.

[23] Mukerjee E.V., Collins S.D., Isseroff R.R., Smith R.L. (2004). Microneedle array for transdermal biological fluid extraction and in situ analysis. Sens. Actuators A Phys..

[24] Wang P.M., Cornwell M., Prausnitz M.R. (2005). Minimally invasive extraction of dermal interstitial fluid for glucose monitoring using microneedles. Diabetes Technol. Ther..

[25] Fernandes D. (2005). Minimally invasive percutaneous collagen induction. Oral Maxillofac. Surg. Clin..

[26] Zaid Alkilani A., McCrudden M.T., Donnelly R.F. (2015). Transdermal drug delivery: Innovative pharmaceutical developments based on disruption of the barrier properties of the stratum corneum. Pharmaceutics.

[27] Sabri A.H., Kim Y., Marlow M., Scurr D.J., Segal J., Banga A.K., Lee J.B. (2020). Intradermal and transdermal drug delivery using microneedles–Fabrication, performance evaluation and application to lymphatic delivery. Adv. Drug Deliv. Rev..

[28] García-López E., Siller H.R., Rodríguez C.A. (2018). Study of the fabrication of AISI 316L microneedle arrays. Procedia Manuf..

[29] Kathuria H., Kang K., Cai J., Kang L. (2020). Rapid microneedle fabrication by heating and photolithography. Int. J. Pharm..

[30] Li Y., Aoude H. (2019). Blast response of beams built with high-strength concrete and high-strength ASTM A1035 bars. Int. J. Impact Eng..

[31] Nejad H.R., Sadeqi A., Kiaee G., Sonkusale S. (2018). Low-cost and cleanroom-free fabrication of microneedles. Microsyst. Nanoeng..

[32] Chen H., Wu B., Zhang M., Yang P., Yang B., Qin W., Wu C. (2019). A novel scalable fabrication process for the production of dissolving microneedle arrays. Drug Deliv. Transl. Res..

[33] Donnelly R.F., Majithiya R., Singh T.R.R., Morrow D.I., Garland M.J., Demir Y.K., Woolfson A.D. (2011). Design, optimization and characterisation of polymeric microneedle arrays prepared by a novel laser-based micromoulding technique. Pharm. Res..

[34] Ligon S.C., Liska R., Stampfl J., Gurr M., Mülhaupt R. (2017). Polymers for 3D printing and customized additive manufacturing. Chem. Rev..

[35] Chen Z., Ren L., Li J., Yao L., Chen Y., Liu B., Jiang L. (2018). Rapid fabrication of microneedles using magnetorheological drawing lithography. Acta Biomater..

[36] Lim J., Tahk D., Yu J., Min D.H., Jeon N.L. (2018). Design rules for a tunable merged-tip microneedle. Microsyst. Nanoeng..

[37] Economidou S.N., Lamprou D.A., Douroumis D. (2018). 3D printing applications for transdermal drug delivery. Int. J. Pharm..

[38] Monzón M.D., Ortega Z., Martínez A., Ortega F. (2015). Standardization in additive manufacturing: Activities carried out by international organizations and projects. Int. J. Adv. Manuf. Technol..

[39] Gittard S.D., Ovsianikov A., Monteiro-Riviere N.A., Lusk J., Morel P., Minghetti P., Narayan R.J. (2009). Fabrication of polymer microneedles using a two-photon polymerization and micromolding process. J. Diabetes Sci. Technol..

[40] Goole J., Amighi K. (2016). 3D printing in pharmaceutics: A new tool for designing customized drug delivery systems. Int. J. Pharm..

[41] Bakhshinejad A., D’souza R.M. A brief comparison between available bio-printing methods. Proceedings of the 2015 IEEE Great Lakes Biomedical Conference (GLBC).

[42] Park S.A., Lee S.J., Lim K.S., Bae I.H., Lee J.H., Kim W.D., Park J.K. (2015). In vivo evaluation and characterization of a bio-absorbable drug-coated stent fabricated using a 3D-printing system. Mater. Lett..

[43] Eltorai A.E., Nguyen E., Daniels A.H. (2015). Three-dimensional printing in orthopedic surgery. Orthopedics.

[44] Tahayeri A., Morgan M., Fugolin A.P., Bompolaki D., Athirasala A., Pfeifer C.S., Bertassoni L.E. (2018). 3D printed versus conventionally cured provisional crown and bridge dental materials. Dent. Mater..

[45] Fan D., Li Y., Wang X., Zhu T., Wang Q., Cai H., Liu Z. (2020). Progressive 3D printing technology and its application in medical materials. Front. Pharmacol..

[46] Olowe M., Parupelli S.K., Desai S. (2022). A Review of 3D-Printing of Microneedles. Pharmaceutics.

[47] Yao W., Li D., Zhao Y., Zhan Z., Jin G., Liang H., Yang R. (2019). 3D printed multi-functional hydrogel microneedles based on high-precision digital light processing. Micromachines.

[48] Chia H.N., Wu B.M. (2015). Recent advances in 3D printing of biomaterials. J. Biol. Eng..

[49] Ngo T.D., Kashani A., Imbalzano G., Nguyen K.T., Hui D. (2018). Additive manufacturing (3D printing): A review of materials, methods, applications and challenges. Compos. Part B Eng..

[50] Zuniga J.M., Cortes A. (2020). The role of additive manufacturing and antimicrobial polymers in the COVID-19 pandemic. Expert Rev. Med. Devices.

[51] Kazi Marzuka S., Kulsum J.U. (2016). 3D Printing: A new avenue in pharmaceuticals. World J. Pharm. Res..

[52] You S., Li J., Zhu W., Yu C., Mei D., Chen S. (2018). Nanoscale 3D printing of hydrogels for cellular tissue engineering. J. Mater. Chem. B.

[53] Chiang H., Yu M., Aksit A., Wang W., Stern-Shavit S., Kysar J.W., Lalwani A.K. (2020). 3D-printed microneedles create precise perforations in human round window membrane in situ. Otol. Neurotol. Off. Publ. Am. Otol. Soc. Am. Neurotol. Soc. Eur. Acad. Otol. Neurotol..

[54] Aimar A., Palermo A., Innocenti B. (2019). The role of 3D printing in medical applications: A state of the art. J. Healthc. Eng..

[55] Au A.K., Huynh W., Horowitz L.F., Folch A. (2016). 3D-printed microfluidics. Angew. Chem. Int. Ed..

[56] Douroumis D. (2019). 3D printing of pharmaceutical and medical applications: A new era. Pharm. Res..

[57] Knowlton S., Yu C.H., Jain N., Ghiran I.C., Tasoglu S. (2015). Smart-phone based magnetic levitation for measuring densities. PLoS ONE.

[58] Yenilmez B., Knowlton S., Tasoglu S. (2016). Self-contained handheld magnetic platform for point of care cytometry in biological samples. Adv. Mater. Technol..

[59] Samiei N. (2020). Recent trends on applications of 3D printing technology on the design and manufacture of pharmaceutical oral formulation: A mini review. Beni-Suef Univ. J. Basic Appl. Sci..

[60] Pardeike J., Strohmeier D.M., Schrödl N., Voura C., Gruber M., Khinast J.G., Zimmer A. (2011). Nanosuspensions as advanced printing ink for accurate dosing of poorly soluble drugs in personalized medicines. Int. J. Pharm..

[61] Prasad L.K., Smyth H. (2016). 3D Printing technologies for drug delivery: A review. Drug Dev. Ind. Pharm..

[62] What Are the Types of 3D Printers and What Can They Do?. https://www.hubs.com/knowledge-base/types-of-3d-printing.

[63] Dawood A., Marti B.M., Sauret-Jackson V., Darwood A. (2015). 3D printing in dentistry. Br. Dent. J..

[64] Systèmes D. (2018). Introduction to 3D Printing–Additive Processes. Dassault Systèmes.

[65] Gibson I., Rosen D., Stucker B., Khorasani M., Rosen D., Stucker B., Khorasani M. (2021). Additive Manufacturing Technologies.

[66] Norman J., Madurawe R.D., Moore C.M., Khan M.A., Khairuzzaman A. (2017). A new chapter in pharmaceutical manufacturing: 3D-printed drug products. Adv. Drug Deliv. Rev..

[67] Xing J.F., Zheng M.L., Duan X.M. (2015). Two-photon polymerization microfabrication of hydrogels: An advanced 3D printing technology for tissue engineering and drug delivery. Chem. Soc. Rev..

[68] Aramian A., Razavi S.M.J., Sadeghian Z., Berto F. (2020). A review of additive manufacturing of cermets. Addit. Manuf..

[69] Brunello G., Sivolella S., Meneghello R., Ferroni L., Gardin C., Piattelli A., Bressan E. (2016). Powder-based 3D printing for bone tissue engineering. Biotechnol. Adv..

[70] Stefaniak A.B., Bowers L.N., Knepp A.K., Luxton T.P., Peloquin D.M., Baumann E.J., Virji M.A. (2019). Particle and vapor emissions from vat polymerization desktop-scale 3-dimensional printers. J. Occup. Environ. Hyg..

[71] Graham A.D., Olof S.N., Burke M.J., Armstrong J.P., Mikhailova E.A., Nicholson J.G., Bayley H. (2017). High-resolution patterned cellular constructs by droplet-based 3D printing. Sci. Rep..

[72] Taylor S.L., Ibeh A.J., Jakus A.E., Shah R.N., Dunand D.C. (2018). NiTi-Nb micro-trusses fabricated via extrusion-based 3D-printing of powders and transient-liquid-phase sintering. Acta Biomater..

[73] Jamróz W., Szafraniec J., Kurek M., Jachowicz R. (2018). 3D printing in pharmaceutical and medical applications–recent achievements and challenges. Pharm. Res..

[74] Iancu C., Iancu D., Stăncioiu A. (2010). From CAD model to 3D print via “STL” file format. Fiability Durab. Fiabil. Durabilitate.

[75] Quan H., Zhang T., Xu H., Luo S., Nie J., Zhu X. (2020). Photo-curing 3D printing technique and its challenges. Bioact. Mater..

[76] Park B.J., Choi H.J., Moon S.J., Kim S.J., Bajracharya R., Min J.Y., Han H.K. (2019). Pharmaceutical applications of 3D printing technology: Current understanding and future perspectives. J. Pharm. Investig..

[77] Carneiro O.S., Silva A.F., Gomes R. (2015). Fused deposition modeling with polypropylene. Mater. Des..

[78] Mohamed O.A., Masood S.H., Bhowmik J.L. (2015). Optimization of fused deposition modeling process parameters: A review of current research and future prospects. Adv. Manuf..

[79] Camposeco-Negrete C. (2020). Optimization of printing parameters in fused deposition modeling for improving part quality and process sustainability. Int. J. Adv. Manuf. Technol..

[80] Luzuriaga M.A., Berry D.R., Reagan J.C., Smaldone R.A., Gassensmith J.J. (2018). Biodegradable 3D printed polymer microneedles for transdermal drug delivery. Lab Chip.

[81] Tang T.O., Holmes S., Dean K., Simon G.P. (2020). Design and fabrication of transdermal drug delivery patch with milliprojections using material extrusion 3D printing. J. Appl. Polym. Sci..

[82] Derakhshandeh H., Aghabaglou F., McCarthy A., Mostafavi A., Wiseman C., Bonick Z., Tamayol A. (2020). A wirelessly controlled smart bandage with 3D-printed miniaturized needle arrays. Adv. Funct. Mater..

[83] Kim H., Han S., Seo Y. (2020). Novel Dual-Curing Process for a Stereolithographically Printed Part Triggers a Remarkably Improved Interlayer Adhesion and Excellent Mechanical Properties. Langmuir.

[84] Economidou S.N., Uddin M.J., Marques M.J., Douroumis D., Sow W.T., Li H., Podoleanu A. (2021). A novel 3D printed hollow microneedle microelectromechanical system for controlled, personalized transdermal drug delivery. Addit. Manuf..

[85] Cekic A., Begic-Hajdarevic D., Cohodar M., Muhamedagic K., Osmanlic M. (2019). Optimization of stereolithography and fused deposition modeling process parameters. Ann. DAAAM Proc..

[86] Zhu S., Chen P., Chen Y., Li M., Chen C., Lu H. (2020). 3D-printed extracellular matrix/polyethylene glycol diacrylate hydrogel incorporating the anti-inflammatory phytomolecule honokiol for regeneration of osteochondral defects. Am. J. Sports Med..

[87] Miller P.R., Gittard S.D., Edwards T.L., Lopez D.M., Xiao X., Wheeler D.R., Narayan R.J. (2011). Integrated carbon fiber electrodes within hollow polymer microneedles for transdermal electrochemical sensing. Biomicrofluidics.

[88] Gittard S.D., Miller P.R., Jin C., Martin T.N., Boehm R.D., Chisholm B.J., Narayan R.J. (2011). Deposition of antimicrobial coatings on microstereolithography-fabricated microneedles. Jom.

[89] Mohamed M.G., Kumar H., Wang Z., Martin N., Mills B., Kim K. (2019). Rapid and inexpensive fabrication of multi-depth microfluidic device using high-resolution LCD stereolithographic 3D printing. J. Manuf. Mater. Process..

[90] Xenikakis I., Tsongas K., Tzimtzimis E.K., Zacharis C.K., Theodoroula N., Kalogianni E.P., Fatouros D.G. (2021). Fabrication of hollow microneedles using liquid crystal display (LCD) vat polymerization 3D printing technology for transdermal macromolecular delivery. Int. J. Pharm..

[91] Balli J., Kumpaty S., Anewenter V. (2017). Continuous liquid interface production of 3D objects: An unconventional technology and its challenges and opportunities. ASME Int. Mech. Eng. Congr. Expo..

[92] Wang H., Zhang W., Ladika D., Yu H., Gailevičius D., Wang H., Yang J.K. (2023). Two-Photon Polymerization Lithography for Optics and Photonics: Fundamentals, Materials, Technologies, and Applications. Adv. Funct. Mater..

[93] Aksit A., Arteaga D.N., Arriaga M., Wang X., Watanabe H., Kasza K.E., Kysar J.W. (2018). In-vitro perforation of the round window membrane via direct 3-D printed microneedles. Biomed. Microdevices.

[94] Plamadeala C., Gosain S.R., Hischen F., Buchroithner B., Puthukodan S., Jacak J., Heitz J. (2020). Bio-inspired microneedle design for efficient drug/vaccine coating. Biomed. Microdevices.

[95] Szeto B., Aksit A., Valentini C., Yu M., Werth E.G., Goeta S., Lalwani A.K. (2021). Novel 3D-printed hollow microneedles facilitate safe, reliable, and informative sampling of perilymph from guinea pigs. Hear. Res..

[96] Doraiswamy A., Jin C., Narayan R.J., Mageswaran P., Mente P., Modi R., Chichkov B. (2006). Two photon induced polymerization of organic–inorganic hybrid biomaterials for microstructured medical devices. Acta Biomater..

[97] Ovsianikov A., Chichkov B., Mente P., Monteiro-Riviere N.A., Doraiswamy A., Narayan R.J. (2007). Two photon polymerization of polymer–ceramic hybrid materials for transdermal drug delivery. Int. J. Appl. Ceram. Technol..

[98] Gittard S.D., Ovsianikov A., Chichkov B.N., Doraiswamy A., Narayan R.J. (2010). Two-photon polymerization of microneedles for transdermal drug delivery. Expert Opin. Drug Deliv..

[99] Jang B.Z., Ma E. (1986). Method and Apparatus for Producing Parts by Selective Snterng. U.S. Patent.

[100] Mazzoli A. (2013). Selective laser sintering in biomedical engineering. Med. Biol. Eng. Comput..

[101] Beg S., Almalki W.H., Malik A., Farhan M., Aatif M., Rahman Z., Rahman M. (2020). 3D printing for drug delivery and biomedical applications. Drug Discov. Today.

[102] Konieczny B., Szczesio-Wlodarczyk A., Sokolowski J., Bociong K. (2020). Challenges of Co–Cr alloy additive manufacturing methods in dentistry—The current state of knowledge (systematic review). Materials.

[103] Rahmati S. (2014). 10.12. Direct Rapid Tooling. Compr. Mater. Process..

[104] Charoo N.A., Barakh Ali S.F., Mohamed E.M., Kuttolamadom M.A., Ozkan T., Khan M.A., Rahman Z. (2020). Selective laser sintering 3D printing–an overview of the technology and pharmaceutical applications. Drug Dev. Ind. Pharm..

[105] Moallemi N., Li R., Mehravaran K. (2016). Breakup of capillary jets with different disturbances. Phys. Fluids.

[106] Gross B.C., Erkal J.L., Lockwood S.Y., Chen C., Spence D.M. (2014). Evaluation of 3D printing and its potential impact on biotechnology and the chemical sciences. Anal. Chem..

[107] Lesch A., Cortés-Salazar F., Bassetto V.C., Amstutz V., Girault H.H. (2015). Inkjet printing meets electrochemical. energy conversion. Chimia.

[108] George E., Liacouras P., Rybicki F.J., Mitsouras D. (2017). Measuring and establishing the accuracy and reproducibility of 3D printed medical models. Radiographics.

[109] Vithani K., Goyanes A., Jannin V., Basit A.W., Gaisford S., Boyd B.J. (2019). An overview of 3D printing technologies for soft materials and potential opportunities for lipid-based drug delivery systems. Pharm. Res..

[110] Awad A., Trenfield S.J., Goyanes A., Gaisford S., Basit A.W. (2018). Reshaping drug development using 3D printing. Drug Discov. Today.

[111] Lay C.L., Koh C.S.L., Lee Y.H., Phan-Quang G.C., Sim H.Y.F., Leong S.X., Ling X.Y. (2020). Two-photon-assisted polymerization and reduction: Emerging formulations and applications. ACS Appl. Mater. Interfaces.

[112] Dabbagh S.R., Sarabi M.R., Rahbarghazi R., Sokullu E., Yetisen A.K., Tasoglu S. (2021). 3D-printed microneedles in biomedical applications. Iscience.

[113] Yang Q., Zhong W., Xu L., Li H., Yan Q., She Y., Yang G. (2021). Recent progress of 3D-printed microneedles for transdermal drug delivery. Int. J. Pharm..

[114] Ambrosi A., Pumera M. (2016). 3D-printing technologies for electrochemical applications. Chem. Soc. Rev..

[115] Jacob S., Nair A.B., Shah J. (2020). Emerging role of nanosuspensions in drug delivery systems. Biomater. Res..

[116] Gardan J. (2016). Additive manufacturing technologies: State of the art and trends. Int. J. Prod. Res..

[117] Zhu C., Liu T., Qian F., Chen W., Chandrasekaran S., Yao B., Li Y. (2017). 3D printed functional nanomaterials for electrochemical energy storage. Nano Today.

[118] Zhang Y., Brown K., Siebenaler K., Determan A., Dohmeier D., Hansen K. (2012). Development of lidocaine-coated microneedle product for rapid, safe, and prolonged local analgesic action. Pharm. Res..

[119] Chen X., Kask A.S., Crichton M.L., McNeilly C., Yukiko S., Dong L., Kendall M.A. (2010). Improved DNA vaccination by skin-targeted delivery using dry-coated densely-packed microprojection arrays. J. Control. Release.

[120] Gratieri T., Alberti I., Lapteva M., Kalia Y.N. (2013). Next generation intra-and transdermal therapeutic systems: Using non-and minimally-invasive technologies to increase drug delivery into and across the skin. Eur. J. Pharm. Sci..

[121] Larraneta E., Lutton R.E., Woolfson A.D., Donnelly R.F. (2016). Microneedle arrays as transdermal and intradermal drug delivery systems: Materials science, manufacture and commercial development. Mater. Sci. Eng. R Rep..

[122] Lee H.J., Son M.J., Ahn J., Oh S.J., Lee M., Kim A., Chung K.S. (2017). Elasticity-based development of functionally enhanced multicellular 3D liver encapsulated in hybrid hydrogel. Acta Biomater..

[123] Chen H.J., Miller P., Shuler M.L. (2018). A pumpless body-on-a-chip model using a primary culture of human intestinal cells and a 3D culture of liver cells. Lab Chip.

[124] Kim B.S., Kwon Y.W., Kong J.S., Park G.T., Gao G., Han W., Cho D.W. (2018). 3D cell printing of in vitro stabilized skin model and in vivo pre-vascularized skin patch using tissue-specific extracellular matrix bioink: A step towards advanced skin tissue engineering. Biomaterials.

[125] Costa E.C., Moreira A.F., de Melo-Diogo D., Gaspar V.M., Carvalho M.P., Correia I.J. (2016). 3D tumor spheroids: An overview on the tools and techniques used for their analysis. Biotechnol. Adv..

[126] Wang S.J., Jiang D., Zhang Z.Z., Chen Y.R., Yang Z.D., Zhang J.Y., Yu J.K. (2019). Biomimetic nanosilica–collagen scaffolds for in situ bone regeneration: Toward a cell-free, one-step surgery. Adv. Mater..

[127] Heidari Keshel S., Rostampour M., Khosropour G., Bandbon B.A., Baradaran-Rafii A., Biazar E. (2016). Derivation of epithelial-like cells from eyelid fat-derived stem cells in thermosensitive hydrogel. J. Biomater. Sci. Polym. Ed..

[128] Zopf D.A., Flanagan C.L., Wheeler M., Hollister S.J., Green G.E. (2014). Treatment of severe porcine tracheomalacia with a 3-dimensionally printed, bioresorbable, external airway splint. JAMA Otolaryngol. Head Neck Surg..

[129] VanKoevering K.K., Morrison R.J., Prabhu S.P., Torres M.F.L., Mychaliska G.B., Treadwell M.C., Green G.E. (2015). Antenatal three-dimensional printing of aberrant facial anatomy. Pediatrics.

[130] Jacobs C.A., Lin A.Y. (2017). A new classification of three-dimensional printing technologies: Systematic review of three-dimensional printing for patient-specific craniomaxillofacial surgery. Plast. Reconstr. Surg..

[131] Legocki A.T., Duffy-Peter A., Scott A.R. (2017). Benefits and limitations of entry-level 3-dimensional printing of maxillofacial skeletal models. JAMA Otolaryngol. Head Neck Surg..

[132] Vukicevic M., Mosadegh B., Min J.K., Little S.H. (2017). Cardiac 3D printing and its future directions. JACC Cardiovasc. Imaging.

[133] Olivieri L.J., Krieger A., Loke Y.H., Nath D.S., Kim P.C., Sable C.A. (2015). Three-dimensional printing of intracardiac defects from three-dimensional echocardiographic images: Feasibility and relative accuracy. J. Am. Soc. Echocardiogr..

[134] Lind J.U., Busbee T.A., Valentine A.D., Pasqualini F.S., Yuan H., Yadid M., Parker K.K. (2017). Instrumented cardiac microphysiological devices via multimaterial three-dimensional printing. Nat. Mater..

[135] Lau I.W.W., Liu D., Xu L., Fan Z., Sun Z. (2018). Clinical value of patient-specific three-dimensional printing of congenital heart disease: Quantitative and qualitative assessments. PLoS ONE.

[136] Mahmood F., Owais K., Taylor C., Montealegre-Gallegos M., Manning W., Matyal R., Khabbaz K.R. (2015). Three-dimensional printing of mitral valve using echocardiographic data. JACC Cardiovasc. Imaging.

[137] Katstra W.E., Palazzolo R.D., Rowe C.W., Giritlioglu B., Teung P., Cima M.J. (2000). Oral dosage forms fabricated by Three Dimensional Printing™. J. Control. Release.

[138] Rowe C.W., Katstra W.E., Palazzolo R.D., Giritlioglu B., Teung P., Cima M.J. (2000). Multimechanism oral dosage forms fabricated by three dimensional printing™. J. Control. Release.

[139] Varghese R., Salvi S., Sood P., Karsiya J., Kumar D. (2022). Recent advancements in additive manufacturing techniques employed in the pharmaceutical industry: A bird–s eye view. Ann. 3D Print. Med..

[140] Goyanes A., Buanz A.B., Hatton G.B., Gaisford S., Basit A.W. (2015). 3D printing of modified-release aminosalicylate (4-ASA and 5-ASA) tablets. Eur. J. Pharm. Biopharm..

[141] Kim C.J. (1995). Compressed donut-shaped tablets with zero-order release kinetics. Pharm. Res..

[142] Sundy E., Danckwerts M.P. (2004). A novel compression-coated doughnut-shaped tablet design for zero-order sustained release. Eur. J. Pharm. Sci..

[143] Halliday A.J., Moulton S.E., Wallace G.G., Cook M.J. (2012). Novel methods of antiepileptic drug delivery—Polymer-based implants. Adv. Drug Deliv. Rev..

[144] Lin S., Chao P.Y., Chien Y.W., Sayani A., Kumar S., Mason M., Monkhouse D. (2001). In vitro and in vivo evaluations of biodegradable implants for hormone replacement therapy: Effect of system design and PK-PD relationship. AAPS PharmSciTech.

[145] Huang W., Zheng Q., Sun W., Xu H., Yang X. (2007). Levofloxacin implants with predefined microstructure fabricated by three-dimensional printing technique. Int. J. Pharm..

[146] Wu W., Zheng Q., Guo X., Huang W. (2009). The controlled-releasing drug implant based on the; three dimensional printing technology: Fabrication and properties of drug releasing in vivo. J. Wuhan Univ. Technol.-Mater. Sci. Ed..

[147] Khan W., Farah S., Domb A.J. (2012). Drug eluting stents: Developments and current status. J. Control. Release.

[148] Tarcha P.J., Verlee D., Hui H.W., Setesak J., Antohe B., Radulescu D., Wallace D. (2007). The application of ink-jet technology for the coating and loading of drug-eluting stents. Ann. Biomed. Eng..

[149] Liao C., Anderson W., Antaw F., Trau M. (2019). Two-photon nanolithography of tailored hollow three-dimensional microdevices for biosystems. ACS Omega.

[150] Milewski M., Brogden N.K., Stinchcomb A.L. (2010). Current aspects of formulation efforts and pore lifetime related to microneedle treatment of skin. Expert Opin. Drug Deliv..

[151] Wang Q.L., Ren J.W., Chen B.Z., Jin X., Zhang C.Y., Guo X.D. (2018). Effect of humidity on mechanical properties of dissolving microneedles for transdermal drug delivery. J. Ind. Eng. Chem..

[152] Wang P.C., Wester B.A., Rajaraman S., Paik S.J., Kim S.H., Allen M.G. Hollow polymer microneedle array fabricated by photolithography process combined with micro molding technique. In Proceedings of 2009 Annual International Conference of the IEEE Engineering in Medicine and Biology Society.

[153] Bhatnagar S., Gadeela P.R., Thathireddy P., Venuganti V.V.K. (2019). Microneedle-based drug delivery: Materials of construction. J. Chem. Sci..

[154] Ali R., Mehta P., Arshad M.S., Kucuk I., Chang M.W., Ahmad Z. (2020). Transdermal microneedles—A materials perspective. AAPS Pharmscitech.

[155] Gupta J., Felner E.I., Prausnitz M.R. (2009). Minimally invasive insulin delivery in subjects with type 1 diabetes using hollow microneedles. Diabetes Technol. Ther..

[156] Sammoura F., Kang J., Heo Y.M., Jung T., Lin L. (2007). Polymeric microneedle fabrication using a microinjection molding technique. Microsyst. Technol..

[157] Davis S.P., Martanto W., Allen M.G., Prausnitz M.R. (2005). Hollow metal microneedles for insulin delivery to diabetic rats. IEEE Trans. Biomed. Eng..

[158] Banga A.K. (2011). Transdermal and Intradermal Delivery of Therapeutic Agents: Application of Physical Technologie.

[159] Nagarkar R., Singh M., Nguyen H.X., Jonnalagadda S. (2020). A review of recent advances in microneedle technology for transdermal drug delivery. J. Drug Deliv. Sci. Technol..

[160] Sirbubalo M., Tucak A., Muhamedagic K., Hindija L., Rahić O., Hadžiabdić J., Vranić E. (2021). 3D printing—A “Touch-Button” approach to manufacture microneedles for transdermal drug delivery. Pharmaceutics.

[161] Takeuchi K., Takama N., Kim B., Sharma K., Paul O., Ruther P. (2019). Microfluidic chip to interface porous microneedles for ISF collection. Biomed. Microdevices.

[162] Yeung C., Chen S., King B., Lin H., King K., Akhtar F., Emaminejad S. (2019). A 3D-printed microfluidic-enabled hollow microneedle architecture for transdermal drug delivery. Biomicrofluidics.

[163] Gill H.S., Prausnitz M.R. (2007). Coated microneedles for transdermal delivery. J. Control. Release.

[164] Kaur M., Ita K.B., Popova I.E., Parikh S.J., Bair D.A. (2014). Microneedle-assisted delivery of verapamil hydrochloride and amlodipine besylate. Eur. J. Pharm. Biopharm..

[165] Jung P.G., Lee T.W., Oh D.J., Hwang S.J., Jung I., Lee S., Ko J. (2008). Nickel microneedles fabricated by sequential copper and nickel electroless plating and copper chemical wet etching. Sens. Mater.

[166] Hong X., Wu Z., Chen L., Wu F., Wei L., Yuan W. (2014). Hydrogel microneedle arrays for transdermal drug delivery. Nano-Micro Lett..

[167] Van Der Maaden K., Jiskoot W., Bouwstra J. (2012). Microneedle technologies for (trans) dermal drug and vaccine delivery. J. Control. Release.

[168] Larrañeta E., McCrudden M.T., Courtenay A.J., Donnelly R.F. (2016). Microneedles: A new frontier in nanomedicine delivery. Pharm. Res..

[169] Yang Y., Kalluri H., Banga A.K. (2011). Effects of chemical and physical enhancement techniques on transdermal delivery of cyanocobalamin (vitamin B12) in vitro. Pharmaceutics.

[170] Gill H.S., Prausnitz M.R. (2007). Coating formulations for microneedles. Pharm. Res..

[171] Fu J., Yu X., Jin Y. (2018). 3D printing of vaginal rings with personalized shapes for controlled release of progesterone. Int. J. Pharm..

[172] Fukushima K., Ise A., Morita H., Hasegawa R., Ito Y., Sugioka N., Takada K. (2011). Two-layered dissolving microneedles for percutaneous delivery of peptide/protein drugs in rats. Pharm. Res..

[173] Zhao X., Coulman S.A., Hanna S.J., Wong F.S., Dayan C.M., Birchall J.C. (2017). Formulation of hydrophobic peptides for skin delivery via coated microneedles. J. Control. Release.

[174] Kapoor Y., Milewski M., Dick L., Zhang J., Bothe J.R., Gehrt M., Smith R. (2020). Coated microneedles for transdermal delivery of a potent pharmaceutical peptide. Biomed. Microdevices.

[175] Marshall S., Sahm L.J., Moore A.C. (2016). The success of microneedle-mediated vaccine delivery into skin. Hum. Vaccines Immunother..

[176] Kim Y.C., Quan F.S., Yoo D.G., Compans R.W., Kang S.M., Prausnitz M.R. (2009). Improved influenza vaccination in the skin using vaccine coated microneedles. Vaccine.

[177] Ito Y., Hagiwara E., Saeki A., Sugioka N., Takada K. (2006). Feasibility of microneedles for percutaneous absorption of insulin. Eur. J. Pharm. Sci..

[178] Donnelly R.F., Singh T.R.R., Woolfson A.D. (2010). Microneedle-based drug delivery systems: Microfabrication, drug delivery, and safety. Drug Deliv..

[179] Donnelly R.F., McCrudden M.T., Zaid Alkilani A., Larrañeta E., McAlister E., Courtenay A.J., Woolfson A.D. (2014). Hydrogel-forming microneedles prepared from “super swelling” polymers combined with lyophilised wafers for transdermal drug delivery. PLoS ONE.

[180] Wang F.Y., Chen Y., Huang Y.Y., Cheng C.M. (2021). Transdermal drug delivery systems for fighting common viral infectious diseases. Drug Deliv. Transl. Res..

[181] Camović M., Biščević A., Brčić I., Borčak K., Bušatlić S., Ćenanović N., Vranić E., Badnjevic A., Škrbić R., Gurbeta Pokvić L. (2020). Coated 3d printed PLA microneedles as transdermal drug delivery systems. CMBEBIH 2019: Proceedings of the International Conference on Medical and Biological Engineering.

[182] Sharma M., Mohapatra S.S., Ranjan S., Dasgupta N., Mishra R.K., Thomas S. (2019). Transdermal and intravenous nano drug delivery systems: Present and future. Applications of Targeted nano Drugs and Delivery Systems.

[183] Halder J., Gupta S., Kumari R., Gupta G.D., Rai V.K. (2021). Microneedle array: Applications, recent advances, and clinical pertinence in transdermal drug delivery. J. Pharm. Innov..

[184] Schmidleithner C., Kalaskar D.M., Cvetković D. (2018). Stereolithography. 3D Printing.

[185] Farias C., Lyman R., Hemingway C., Chau H., Mahacek A., Bouzos E., Mobed-Miremadi M. (2018). Three-dimensional (3D) printed microneedles for microencapsulated cell extrusion. Bioengineering.

[186] Economidou S.N., Pere C.P.P., Reid A., Uddin M.J., Windmill J.F., Lamprou D.A., Douroumis D. (2019). 3D printed microneedle patches using stereolithography (SLA) for intradermal insulin delivery. Mater. Sci. Eng. C.

[187] Xenikakis I., Tzimtzimis M., Tsongas K., Andreadis D., Demiri E., Tzetzis D., Fatouros D.G. (2019). Fabrication and finite element analysis of stereolithographic 3D printed microneedles for transdermal delivery of model dyes across human skin in vitro. Eur. J. Pharm. Sci..

[188] Lim S.H., Tiew W.J., Zhang J., Ho P.C.L., Kachouie N.N., Kang L. (2020). Geometrical optimisation of a personalised microneedle eye patch for transdermal delivery of anti-wrinkle small peptide. Biofabrication.

[189] Lu Y., Mantha S.N., Crowder D.C., Chinchilla S., Shah K.N., Yun Y.H., Choi J.W. (2015). Microstereolithography and characterization of poly (propylene fumarate)-based drug-loaded microneedle arrays. Biofabrication.

[190] Mansor N.H.A., Markom M.A., Tan E.S.M.M., Adom A.H. (2019). Design and fabrication of biodegradable microneedle using 3D rapid prototyping printer. J. Phys. Conf. Ser..

[191] Lee P.H., Chung H., Lee S.W., Yoo J., Ko J. (2014). Dimensional accuracy in additive manufacturing processes. ASME 2014 International Manufacturing Science and Engineering Conference.

[192] Lemu H.G., Kurtovic S., Frick J., Laugen B.T. (2012). 3D printing for rapid manufacturing: Study of dimensional and geometrical accuracy. Advances in Production Management Systems, Value Networks: Innovation, Technologies, and Management.

[193] Zha W., Anand S. (2015). Geometric approaches to input file modification for part quality improvement in additive manufacturing. J. Manuf. Process..

[194] Economidou S.N., Pissinato Pere C.P., Okereke M., Douroumis D. (2021). Optimisation of design and manufacturing parameters of 3D printed solid microneedles for improved strength, sharpness, and drug delivery. Micromachines.

[195] Wu M., Zhang Y., Huang H., Li J., Liu H., Guo Z., Lei Y. (2020). Assisted 3D printing of microneedle patches for minimally invasive glucose control in diabetes. Mater. Sci. Eng. C.

[196] Vranić E., Tucak A., Sirbubalo M., Rahić O., Elezović A., Hadžiabdić J. (2020). Microneedle-based sensor systems for real-time continuous transdermal monitoring of analytes in body fluids. CMBEBIH 2019: Proceedings of the International Conference on Medical and Biological Engineering.

[197] Kjar A., Huang Y. (2019). Application of micro-scale 3D printing in pharmaceutics. Pharmaceutics.

[198] Aoyagi S., Izumi H., Fukuda M. (2008). Biodegradable polymer needle with various tip angles and consideration on insertion mechanism of mosquito–s proboscis. Sens. Actuators A Phys..

[199] Li C.G., Lee C.Y., Lee K., Jung H. (2013). An optimized hollow microneedle for minimally invasive blood extraction. Biomed. Microdevices.

[200] Sausse M., Gobillon C., Lambert P. (2013). Microneedle array penetration tests: Understanding the “bed of nails” phenomenon. ONdrugDelivery.

[201] Andersen N.K., Taboryski R. (2017). Drop shape analysis for determination of dynamic contact angles by double sided elliptical fitting method. Meas. Sci. Technol..

[202] van der Maaden K., Sekerdag E., Schipper P., Kersten G., Jiskoot W., Bouwstra J. (2015). Layer-by-layer assembly of inactivated poliovirus and N-trimethyl chitosan on pH-sensitive microneedles for dermal vaccination. Langmuir.

[203] Lutton R.E., Moore J., Larrañeta E., Ligett S., Woolfson A.D., Donnelly R.F. (2015). Microneedle characterisation: The need for universal acceptance criteria and GMP specifications when moving towards commercialisation. Drug Deliv. Transl. Res..

[204] Park J.H., Allen M.G., Prausnitz M.R. (2005). Biodegradable polymer microneedles: Fabrication, mechanics and transdermal drug delivery. J. Control. Release.

[205] Davis S.P., Landis B.J., Adams Z.H., Allen M.G., Prausnitz M.R. (2004). Insertion of microneedles into skin: Measurement and prediction of insertion force and needle fracture force. J. Biomech..

[206] Gittard S.D., Chen B., Xu H., Ovsianikov A., Chichkov B.N., Monteiro-Riviere N.A., Narayan R.J. (2013). The effects of geometry on skin penetration and failure of polymer microneedles. J. Adhes. Sci. Technol..

[207] Yu W., Jiang G., Zhang Y., Liu D., Xu B., Zhou J. (2017). Polymer microneedles fabricated from alginate and hyaluronate for transdermal delivery of insulin. Mater. Sci. Eng. C.

[208] Donnelly R.F., Singh T.R.R., Garland M.J., Migalska K., Majithiya R., McCrudden C.M., Woolfson A.D. (2012). Hydrogel-forming microneedle arrays for enhanced transdermal drug delivery. Adv. Funct. Mater..

[209] Larrañeta E., Moore J., Vicente-Pérez E.M., González-Vázquez P., Lutton R., Woolfson A.D., Donnelly R.F. (2014). A proposed model membrane and test method for microneedle insertion studies. Int. J. Pharm..

[210] Coulman S.A., Birchall J.C., Alex A., Pearton M., Hofer B., O’Mahony C., Považay B. (2011). In vivo, in situ imaging of microneedle insertion into the skin of human volunteers using optical coherence tomography. Pharm. Res..

[211] Applegate B.E., Izatt J.A. (2007). Molecular contrast OCT. Optical Coherence Tomography in Cardiovascular Research.

[212] Virmani R., Burke A.P., Farb A., Kolodgie F.D. (2006). Pathology of the vulnerable plaque. J. Am. Coll. Cardiol..

[213] Moronkeji K., Todd S., Dawidowska I., Barrett S.D., Akhtar R. (2017). The role of subcutaneous tissue stiffness on microneedle performance in a representative in vitro model of skin. J. Control. Release.

[214] Pattani A., McKay P.F., Garland M.J., Curran R.M., Migalska K., Cassidy C.M., Donnelly R.F. (2012). Microneedle mediated intradermal delivery of adjuvanted recombinant HIV-1 CN54gp140 effectively primes mucosal boost inoculations. J. Control. Release.

[215] Loizidou E.Z., Inoue N.T., Ashton-Barnett J., Barrow D.A., Allender C.J. (2016). Evaluation of geometrical effects of microneedles on skin penetration by CT scan and finite element analysis. Eur. J. Pharm. Biopharm..

[216] Kusamori K., Katsumi H., Sakai R., Hayashi R., Hirai Y., Tanaka Y., Yamamoto A. (2016). Development of a drug-coated microneedle array and its application for transdermal delivery of interferon alpha. Biofabrication.

[217] Gupta J., Gupta R., Vanshita (2021). Microneedle technology: An insight into recent advancements and future trends in drug and vaccine delivery. Assay Drug Dev. Technol..

[218] Moss G.P., Gullick D.R., Wilkinson S.C., Moss G.P., Gullick D.R., Wilkinson S.C. (2015). Methods for the Measurement of Percutaneous Absorption. Predictive Methods in Percutaneous Absorption.

[219] Benson H.A., Watkinson A.C. (2012). Topical and Transdermal Drug Delivery: Principles and Practice.

[220] Bronaugh R.L., Stewart R.F. (1985). Methods for in vitro percutaneous absorption studies IV: The flow-through diffusion cell. J. Pharm. Sci..

[221] Cao Y., Tao Y., Zhou Y., Gui S. (2016). Development of sinomenine hydrochloride-loaded polyvinylalcohol/maltose microneedle for transdermal delivery. J. Drug Deliv. Sci. Technol..

[222] Donnelly R.F., Morrow D.I., Fay F., Scott C.J., Abdelghany S., Singh R.R.T., Woolfson A.D. (2010). Microneedle-mediated intradermal nanoparticle delivery: Potential for enhanced local administration of hydrophobic pre-formed photosensitisers. Photodiagnosis Photodyn. Ther..

[223] Flaten G.E., Palac Z., Engesland A., Filipović-Grčić J., Vanić Ž., Škalko-Basnet N. (2015). In vitro skin models as a tool in optimization of drug formulation. Eur. J. Pharm. Sci..

[224] Todo H. (2017). Transdermal permeation of drugs in various animal species. Pharmaceutics.

[225] Dong L., Li Y., Li Z., Xu N., Liu P., Du H., Tao J. (2018). Au nanocage-strengthened dissolving microneedles for chemo-photothermal combined therapy of superficial skin tumors. ACS Appl. Mater. Interfaces.

[226] Topalian S.L., Drake C.G., Pardoll D.M. (2015). Immune checkpoint blockade: A common denominator approach to cancer therapy. Cancer Cell.

[227] Jeanbart L., Swartz M.A. (2015). Engineering opportunities in cancer immunotherapy. Proc. Natl. Acad. Sci. USA.

[228] Chen M.C., Lin Z.W., Ling M.H. (2016). Near-infrared light-activatable microneedle system for treating superficial tumors by combination of chemotherapy and photothermal therapy. ACS Nano.

[229] van der Maaden K., Heuts J., Camps M., Pontier M., van Scheltinga A.T., Jiskoot W., Bouwstra J. (2018). Hollow microneedle-mediated micro-injections of a liposomal HPV E743–63 synthetic long peptide vaccine for efficient induction of cytotoxic and T-helper responses. J. Control. Release.

[230] Tang T., Deng Y., Chen J., Zhao Y., Yue R., Choy K.W., Chung T.K.H. (2016). Local administration of siRNA through microneedle: Optimization, bio-distribution, tumor suppression and toxicity. Sci. Rep..

[231] Uddin M.J., Scoutaris N., Economidou S.N., Giraud C., Chowdhry B.Z., Donnelly R.F., Douroumis D. (2020). 3D printed microneedles for anticancer therapy of skin tumours. Mater. Sci. Eng. C.

[232] Bhatnagar S., Chawla S.R., Kulkarni O.P., Venuganti V.V.K. (2017). Zein microneedles for transcutaneous vaccine delivery: Fabrication, characterization, and in vivo evaluation using ovalbumin as the model antigen. Acs Omega.

[233] Bhatnagar S., Kumari P., Pattarabhiran S.P., Venuganti V.V.K. (2018). Zein microneedles for localized delivery of chemotherapeutic agents to treat breast cancer: Drug loading, release behavior, and skin permeation studies. Aaps Pharmscitech.

[234] Ma Y., Boese S.E., Luo Z., Nitin N., Gill H.S. (2015). Drug coated microneedles for minimally-invasive treatment of oral carcinomas: Development and in vitro evaluation. Biomed. Microdevices.

[235] Zhou Z., Lin H., Li C., Wu Z. (2018). Recent progress of fully synthetic carbohydrate-based vaccine using TLR agonist as build-in adjuvant. Chin. Chem. Lett..

[236] Ding Z., Verbaan F.J., Bivas-Benita M., Bungener L., Huckriede A., van den Berg D.J., Bouwstra J.A. (2009). Microneedle arrays for the transcutaneous immunization of diphtheria and influenza in BALB/c mice. J. Control. Release.

[237] Li G., Badkar A., Nema S., Kolli C.S., Banga A.K. (2009). In vitro transdermal delivery of therapeutic antibodies using maltose microneedles. Int. J. Pharm..

[238] Sullivan S.P., Koutsonanos D.G., del Pilar Martin M., Lee J.W., Zarnitsyn V., Choi S.O., Prausnitz M.R. (2010). Dissolving polymer microneedle patches for influenza vaccination. Nat. Med..

[239] Raphael A.P., Crichton M.L., Falconer R.J., Meliga S., Chen X., Fernando G.J., Kendall M.A. (2016). Formulations for microprojection/microneedle vaccine delivery: Structure, strength and release profiles. J. Control. Release.

[240] De Groot A.M., Platteel A.C., Kuijt N., Van Kooten P.J., Vos P.J., Sijts A.J., Van der Maaden K. (2017). Nanoporous microneedle arrays effectively induce antibody responses against diphtheria and tetanus toxoid. Front. Immunol..

[241] Caudill C., Perry J.L., Iliadis K., Tessema A.T., Lee B.J., Mecham B.S., DeSimone J.M. (2021). Transdermal vaccination via 3D-printed microneedles induces potent humoral and cellular immunity. Proc. Natl. Acad. Sci. USA.

[242] Fabbrocini G., De Vita V., Monfrecola A., De Padova M.P., Brazzini B., Teixeira F., Chu A. (2014). Percutaneous collagen induction: An effective and safe treatment for post-acne scarring in different skin phototypes. J. Dermatol. Treat..

[243] Cachafeiro T., Escobar G., Maldonado G., Cestari T., Corleta O. (2016). Comparison of nonablative fractional erbium laser 1,340 nm and microneedling for the treatment of atrophic acne scars: A randomized clinical trial. Dermatol. Surg..

[244] Kim M., Yang H., Kim H., Jung H., Jung H. (2014). Novel cosmetic patches for wrinkle improvement: Retinyl retinoate-and ascorbic acid-loaded dissolving microneedles. Int. J. Cosmet. Sci..

[245] Prausnitz M.R. (2017). Engineering microneedle patches for vaccination and drug delivery to skin. Annu. Rev. Chem. Biomol. Eng..

[246] Lim S.H., Kathuria H., Amir M.H.B., Zhang X., Duong H.T., Ho P.C.L., Kang L. (2021). High resolution photopolymer for 3D printing of personalised microneedle for transdermal delivery of anti-wrinkle small peptide. J. Control. Release.

[247] Ross S., Scoutaris N., Lamprou D., Mallinson D., Douroumis D. (2015). Inkjet printing of insulin microneedles for transdermal delivery. Drug Deliv. Transl. Res..

[248] Boehm R.D., Miller P.R., Schell W.A., Perfect J.R., Narayan R.J. (2013). Inkjet printing of amphotericin B onto biodegradable microneedles using piezoelectric inkjet printing. Jom.

[249] Boehm R.D., Daniels J., Stafslien S., Nasir A., Lefebvre J., Narayan R.J. (2015). Polyglycolic acid microneedles modified with inkjet-deposited antifungal coatings. Biointerphases.

[250] Uddin M.J., Scoutaris N., Klepetsanis P., Chowdhry B., Prausnitz M.R., Douroumis D. (2015). Inkjet printing of transdermal microneedles for the delivery of anticancer agents. Int. J. Pharm..

[251] El-Sayed N., Vaut L., Schneider M. (2020). Customized fast-separable microneedles prepared with the aid of 3D printing for nanoparticle delivery. Eur. J. Pharm. Biopharm..

[252] Cordeiro A.S., Tekko I.A., Jomaa M.H., Vora L., McAlister E., Volpe-Zanutto F., Donnelly R.F. (2020). Two-photon polymerisation 3D printing of microneedle array templates with versatile designs: Application in the development of polymeric drug delivery systems. Pharm. Res..

[253] Li X., Shan W., Yang Y., Joralmon D., Zhu Y., Chen Y., Chen Y. (2021). Limpet tooth-inspired painless microneedles fabricated by magnetic field-assisted 3D printing. Adv. Funct. Mater..

[254] Johnson A.R., Caudill C.L., Tumbleston J.R., Bloomquist C.J., Moga K.A., Ermoshkin A., DeSimone J.M. (2016). Single-step fabrication of computationally designed microneedles by continuous liquid interface production. PLoS ONE.

[255] Chen Z., Ye R., Yang J., Lin Y., Lee W., Li J., Jiang L. (2019). Rapidly fabricated microneedle arrays using magnetorheological drawing lithography for transdermal drug delivery. ACS Biomater. Sci. Eng..

[256] Chen Z., Li Z., Li J., Liu C., Lao C., Fu Y., He Y. (2019). 3D printing of ceramics: A review. J. Eur. Ceram. Soc..

[257] Han D., Morde R.S., Mariani S., La Mattina A.A., Vignali E., Yang C., Lee H. (2020). 4D printing of a bioinspired microneedle array with backward-facing barbs for enhanced tissue adhesion. Adv. Funct. Mater..

[258] O’Shea J., Prausnitz M.R., Rouphael N. (2021). Dissolvable microneedle patches to enable increased access to vaccines against SARS-CoV-2 and future pandemic outbreaks. Vaccines.

[259] Vaishya R., Javaid M., Khan I.H., Haleem A. (2020). Artificial Intelligence (AI) applications for COVID-19 pandemic. Diabetes Metab. Syndr. Clin. Res. Rev..

